# Macrophages mediate psoriasis via Mincle-dependent mechanism in mice

**DOI:** 10.1038/s41420-023-01444-8

**Published:** 2023-04-28

**Authors:** Rui-zhi Tan, Xia Zhong, Rang-yue Han, Ke-huan Xie, Jian Jia, Ye Yang, Mei Cheng, Chun-yan Yang, Hui-yao Lan, Li Wang

**Affiliations:** 1 Research Center of Intergated Traditional Chinese and Western Medicine, Affiliated Traditional Chinese Medicine Hospital, Southwest Medical University, Luzhou, China; 2 Institute of Integrated Chinese and Western Medicine, Southwest Medical University, Luzhou, China; 3 Department of Orthopaedics, Affiliated Traditional Chinese Medicine Hospital, Southwest Medical University, Luzhou, China; 4 Dermatological Department, Affiliated Traditional Chinese Medicine Hospital, Southwest Medical University, Luzhou, China; 5 Department of Medicine and Therapeutics, Li Ka Shing Institute of Health Sciences, The Chinese University of Hong Kong, Hong Kong, China

**Keywords:** Chronic inflammation, Skin diseases

## Abstract

Psoriasis is currently considered to be an immune and inflammatory disease characterized by massive immune cells infiltration including macrophages. It has been reported that macrophage-inducible C-type lectin (Mincle) is essential to maintain the pro-inflammatory phenotype of M1 macrophages, however, its role and mechanisms in psoriasis remain largely unknown. A model of psoriasis was induced in mice by a daily topical application of imiquimod for 7 days. Role and mechanisms of Mincle in macrophage-mediated psoriasis were investigated in clodronate liposomes induced macrophage depletion mice followed by adoptively transferring with Mincle-expressing or -knockout (KO) macrophages, and in macrophage specific Mincle knockout mice (Mincle^loxp/loxp^/Lyz2-cre^+/+^). Finally, a Mincle neutralizing antibody was employed to the psoriasis mice to reveal the therapeutic potential for psoriasis by targeting Mincle. Mincle was highly expressed by M1 macrophages in the skin lesions of patients and mice with psoriasis. Clodronate liposomes-induced macrophage depletion inhibited psoriasis in mice, which was restored by adoptive transfer with Mincle-expressing macrophages but not by Mincle-KO macrophages. This was further confirmed in macrophage-specific Mincle-KO mice. Mechanistically, macrophages mediated psoriasis via the Mincle-Syk-NF-κB pathway as blocking macrophage Mincle inhibited Syk/NF-κB-driven skin lesions and epidermal injury in vivo and in vitro. We also found that LPS induced Mincle expression by M1 macrophages via the PU.1-dependent mechanism. Most importantly, we revealed that targeting Mincle with a neutralizing antibody significantly improved psoriasis in mice. In summary, our findings demonstrated that macrophages mediate psoriasis in mice via the Mincle-dependent mechanism, targeting Mincle may represent as a novel therapy for psoriasis.

**Figure:**
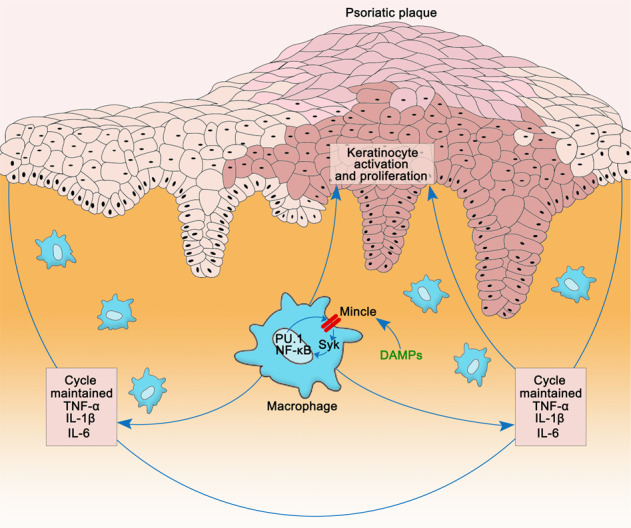
A simplified pathway model of Mincle in macrophage-mediated psoriasis.

A simplified pathway model of Mincle in macrophage-mediated psoriasis.

## Background

Psoriasis is a chronic, papulosquamous skin disease with an incidence range from 0.14% in East Asia to 1.99% in Australia, and the main peak of incidence is around 39 and 50–59 years old [1–4]. The main feature of psoriasis is hereditary, recurrence, and may also concurrent cardiovascular disease, diabetes and arthritis [5, 6]. The current understanding of psoriasis has changed from an epidermal keratinocyte induced disease to an immune-mediated disease [7], however, the specific pathogenesis is not fully explained. Psoriasis severely affects the patient’s physical and mental health as well as quality of life, has become a common disease that threatening public health.

An important feature of psoriasis is sustained inflammation, leading to uncontrollable keratinocyte proliferation and abnormal functional differentiation, accompanied by a large number of immune cell infiltration, such as dendritic cells, macrophages, T cells, and neutrophils, etc [8, 9]. Among them, macrophage exhibit significant plasticity and are able to self-regulate their phenotype and function in response to different surrounding environments [10, 11]. Studies have shown that macrophages are significantly increased in the epidermal tissue of patients with psoriasis, suggesting that macrophage may play an important role in psoriasis [12, 13]. A recent study demonstrated that macrophage, but not granulocytes or alphabeta T lymphocytes contribute to triggering a psoriasis-like skin disease with hyperproliferation and inflammation [14]. Another study also reported that the maintenance of psoriasis skin inflammation mainly depends on the recruitment and activation of macrophage and the release of TNF-α [15]. Results from these suggest a critical role for macrophages in the development of skin psoriasis lesion. Therefore, exploring the mechanism of macrophage-mediated inflammation may be a promising research approach.

Macrophage-inducible C-type lectin (Mincle) is a pattern recognition receptor mainly expressed in macrophages [16]. Mincle can recognize damage associated molecular patterns (DAMPs) and pathogen associated molecular patterns (PAMPs), and is involved in the inflammatory response of macrophage-mediated immune and inflammatory diseases [17–19]. It is now clear that Mincle is activated through the MYD88 of Toll-like receptor signaling pathway [20]. Once being activated, Mincle signals through Syk to further cause the phosphorylation of NF-κB (P-65), thereby enhancing the inflammatory response [21]. An important study showed that Mincle triggers and maintains the phenotype of M1 pro-inflammatory macrophages while inhibiting the activity of M2 anti-inflammatory macrophages to aggravate macrophage-dependent [22]. However, the role and mechanisms of Mincle in macrophage-mediated psoriasis remain unknown.

In this present study we investigated the role of Mincle in macrophage-mediated psoriasis in mice, and revealed mechanisms though which Mince regulated macrophage-dependent psoriasis injury. Finally, we also developed a novel therapy for psoriasis by targeting Mincle in mice.

## Results

### Mincle is up-regulated and correlated with severe skin inflammation in patients with Psoriasis

**Fig. 1:**
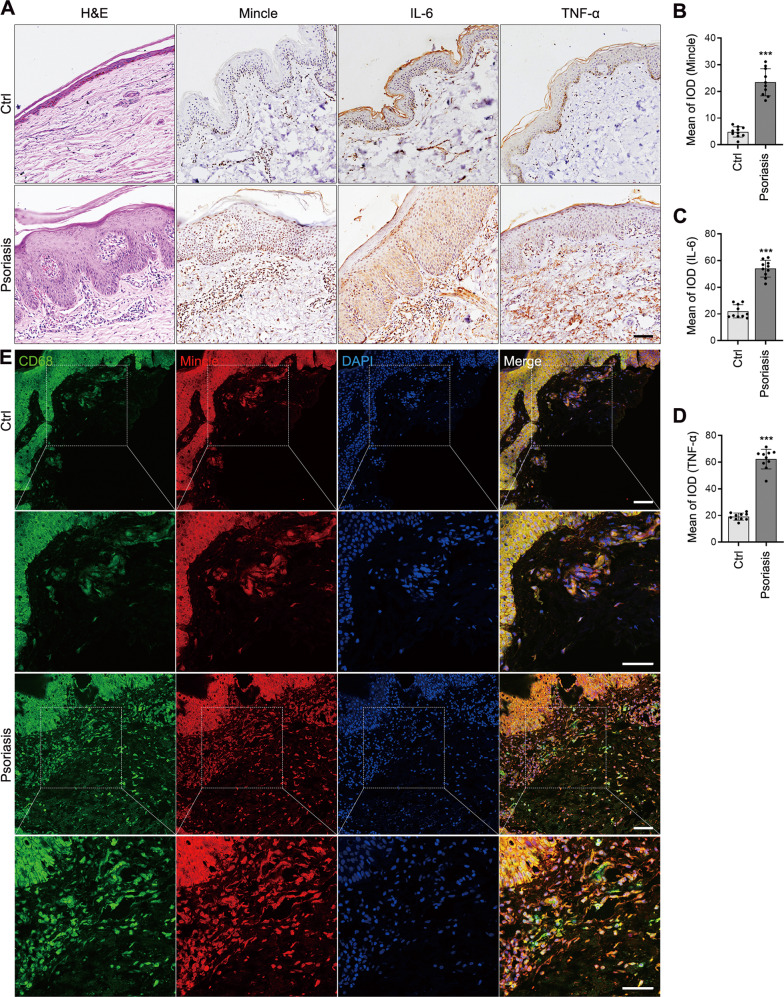
Mincle is up-regulated and correlated with severe skin inflammation in patients with Psoriasis. **A**–**D** Representative images of H&E and immunohistochemical staining showing the skin lesions and expression of Mincle, IL-6 and TNF-α in normal person and patients’ skins. **E** Representative images of immunofluorescence staining showing a markedly increased level of Mincle and CD68 in the skin of psoriasis patients. *n* = 10 in each group. ***P < 0.001 vs. Ctrl.

We first examined Mincle expression and macrophage inflammation in patients with psoriasis in skin biopsy samples. Compared to healthy controls, immunohistochemistry showed that Mincle was strongly expressed in the skin lesions of patients with psoriasis, accompanied by a significant macrophage accumulation and upregulation of inflammatory cytokines (Fig. 1A–D). Two-color immunofluorescence revealed that the majority of Mincle-expressing cells were macrophages as identified by co-expressing CD68 and Mincle (CD68 + Mincle + ) (Fig. 1E), providing a direct evidence for Mincle-expressing macrophages in psoriasis lesions and a rationale for studying the role of Mincle in macrophage-mediated psoriasis.

### Mincle is upregulated with severe skin inflammation in IMQ-induced psoriasis mouse model

**Fig. 2:**
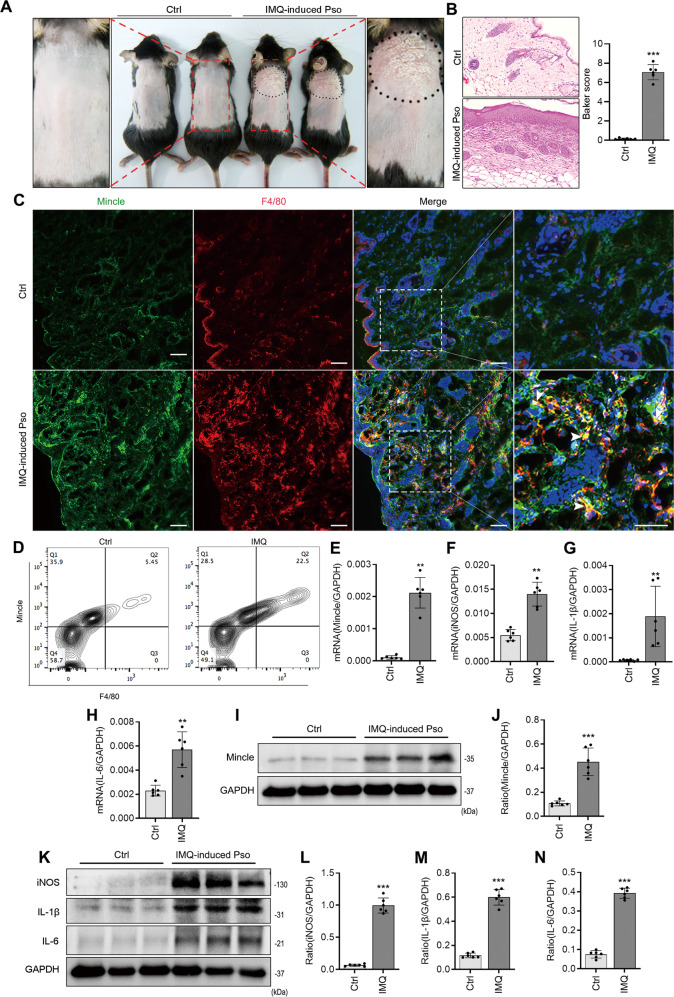
Mincle is upregulated with severe skin inflammation in IMQ-induced psoriasis mouse model. **A** Psoriasis-like skin lesions induced by miquimod. **B** Representative images of H&E staining and baker score showing the skin tissue pathology of Ctrl and IMQ mouse. **C** Representative images of immunofluorescence staining and quantification showing a markedly increased level of Mincle and F4/80 in the skin of psoriasis mouse. **D** Flow cytometry detected the expression of Mincle and F4/80 in cell suspension of skin. **E**–**H** Real-time PCR analyze the mRNA expression of Mincle, iNOS, IL-1β and IL-6 mRNA. **I**, **J** Western blot results showed that Mincle was increased in the skin lesions of psoriasis mice caused by IMQ. **K**–**N** Western blot detected the protein levels of iNOS, IL-1β and IL-6. *n* = 6 in each group. **P < 0.01, ***P < 0.001 vs. Ctrl.

To confirm the pathological observations of Mincle-expressing macrophages in patients with psoriasis, we established a psoriasis-like mouse model by topically applying miquimod (IMQ) to the skin on the back of C57BL mice for 7 consecutive days. As shown in Fig. 2, the symptoms of the erythema, scaling, and thickening of skin were developed in IMQ-treated mice but not in the Ctrl mice (Fig. 2A). The H&E staining showed that IMQ treatment for 7 days dramatically increased the epidermal thickness and infiltration of mononuclear cells (Fig. 2B). Two-color immunofluorescence detected that there were many F4/80+ macrophages infiltrating the skin lesions, of them, most of cells co-expressed Mincle as identified by Mincle + /F4/80+ cells, revealing M1 macrophage phenotype (Fig. 2C). This observation was further confirmed by two-color flow cytometry with the finding of almost all of F4/80+ macrophages co-expressing Mincle (Fig. 2D). Real-time PCR and western blot results also showed that, compared with the normal mice, Mincle mRNA and protein levels were significantly increased in the skin of IMQ-induced psoriasis (Fig. 2E, I, J), which were associated with a marked upregulation pro-inflammatory cytokines iNOS, IL-1β and IL-6 (Fig. 2F–H, K-–), demonstrating that IMQ treatment can induce psoriasis-like skin lesions in mice mimicking psoriasis in patients.

### Depletion of macrophages with clodronate liposomes inhibits IMQ-induced psoriasis in mice, which is restored by adoptive transfer with Mincle-expressing macrophages but not by Mincle-KO macrophages

**Fig. 3:**
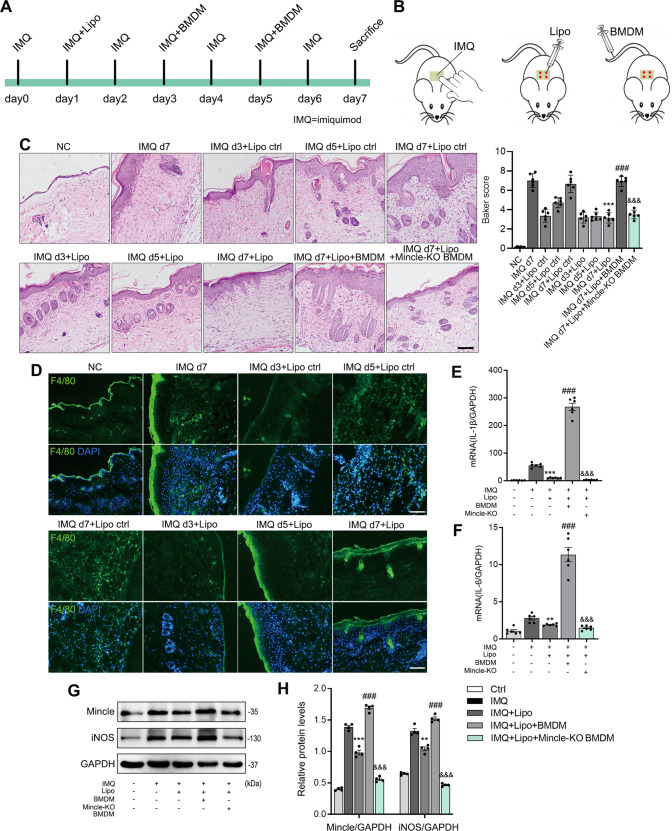
Depletion of macrophages with clodronate liposomes inhibits IMQ-induced psoriasis in mice, which is restored by adoptive transfer with Mincle-expressing macrophages but not by Mincle-KO macrophages. **A** The timeline of injection of lipo and BMDM in psoriasis mice. **B** Schematic diagram of administration in mice. **C** H&E staining and baker score showed the histopathology of mouse skin in each group. **D** Representative images of immunofluorescence staining after removing macropahges from skin by injection of lipo. **E**, **F** The real-time PCR results of IL-1β and IL-6 mRNA levels in each group. **G**, **H** Western blot results showed the protein levels of Mincle and iNOS in each group. *n* = 6 in each group. **P < 0.01, ***P < 0.001 vs. IMQ group; ^###^*P* < 0.001 vs. IMQ + Lipo group; ^&&&^*P* < 0.001 vs. IMQ + Lipo + BMDM group.

In order to determine the effect of Mincle on macrophage-mediated psoriasis injury, one day after IMQ treatment, we injected clodronate liposomes subcutaneously to the mouse skin lesions to deplete the macrophages locally, followed by adoptively transferred via tail vein with Tomato-expressing BMDM (macrophages expressing red fluorescence, Supplementary Fig. 1A) or Mincle-KO BMDM on the 3rd and 5th days after clodronate liposomes administration (Fig. 3A, B). The results demonstrated that tail vein injected Tomato-expressing BMDM can well infiltrate to IMQ-stimulated skin (Supplementary Fig 1B). Immunofluorescence results also showed that, compared with liposomes control, treatment with clodronate liposomes significantly reduced F4/80+ macrophage infiltration and skin lesions on the 5th, and 7th day after IMQ treatment (Fig. 3D). Adoptive transfer with Tomato-expressing BMDMs restored Mincle-expressing macrophage infiltration and inflammatory responses as demonstrated by upregulation of pro-inflammatory cytokines (IL-1β and IL-6) (Fig. 3E, F), Mincle and iNOS (Fig. 3G, H), resulting in the development of severe skin lesion in psoriasis mice (Fig. 3C). In contrast, adoptive transfer with Mincle-KO BMDMs did not increase the severity of skin lesions and inflammation when compared with macrophage-depleting mice (Fig. 3C). All these observations demonstrate a critical role for macrophages in the pathogenesis of psoriasis via the Mincle-dependent mechanism.

### Conditional deletion of Mincle from macrophages inhibits IMQ-induced psoriasis in Mincle^flox/flox^/Lyz-cre^+/+^ mice

**Fig. 4:**
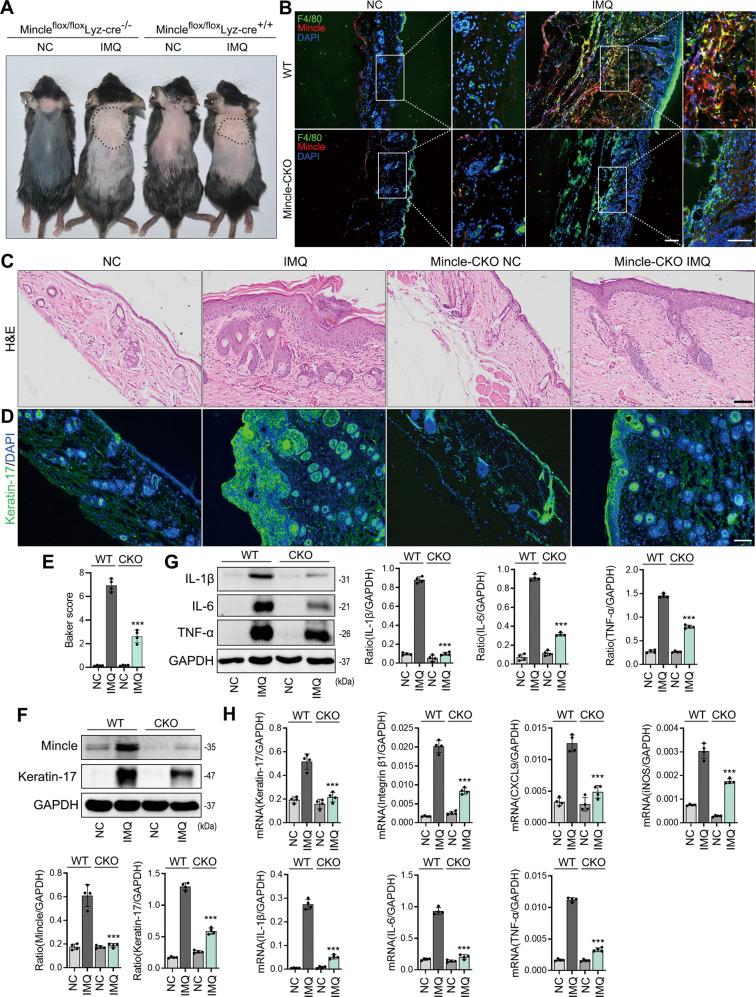
Conditional deletion of Mincle from macrophages inhibits IMQ-induced psoriasis in Mincle^flox/flox^/Lyz-cre^+/+^ mice. **A** Psoriasis-like skin lesions induced by miquimod in WT and Mincle-KO mouse model. **B** Representative images of immunofluorescence staining of F4/80 and Mincle in WT and Mincle-KO mouse model. **C** H&E staining showed the histopathology of skin in WT and Mincle-KO mouse model. **D** Representative images of immunofluorescence staining of keratin-17 in WT and Mincle-KO mouse model. **E** Baker score of H&E staining. **F** Western blot showed the protein levels of Mincle and Keratin-17 in each group. **G** Western blot showed the protein levels of IL-1β, IL-6 and TNF-α in each group. **H** Real-time PCR detected the mRNA expression of Keratin-17, Intergrin β1, CXCL9, iNOS, IL-1β, IL-6 and TNF-α in each group. *n* = 4 in each group. ***P < 0.001 vs. WT-IMQ group.

To further verify the role of Mincle in macrophage-mediated psoriasis, we employed macrophage-specific Mincle KO mice (Mincle^flox/flox^/Lyz-cre^+/+^) to establish a psoriasis model. Compared to Mincle^flox/flox^/Lyz-cre^−/−^ control littermate mice immunofluorescence confirmed the deletion of Mincle-expressing macrophages in the skin lesions and the significant improvement in the severity of psoriasis in Mincle^flox/flox^/Lyz-cre^+/+^ mice with a few Mincle + /F4/80+ macrophages (Fig. 4A–C, E). Immunofluorescence and western analysis revealed that keratin-17 was strongly inhibited after Mincle being deleted from macrophages in Mincle^flox/flox^/Lyz-cre^+/+^ mice (Fig. 4D, F). In addition, the protein levels of inflammatory factor-related protein were also down-regulated (IL-1β, IL-6 and TNF-α) (Fig. 4G). Real-time PCR results further confirmed that the mRNA levels of psoriasis-related factors keratin-17, integrin β1, and CXCL9, as well as the mRNA levels of inflammation-related factors IL-1β, IL-6, TNF-α and iNOS, were strongly inhibited in skin of Mincle^flox/flox^/Lyz-cre^+/+^ mice (Fig. 4H). All these results confirm a pathogenic role for Mincle in macrophage-mediated psoriasis.

### Mincle mediates M1 macrophage activation via the Syk-NF-κB mechanism in vivo and in vitro

**Fig. 5:**
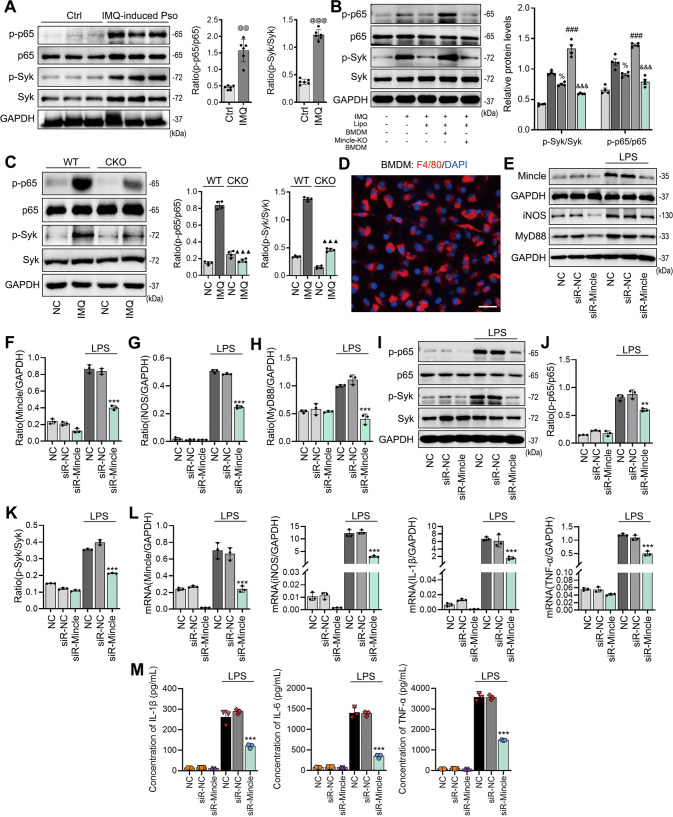
Mincle mediates M1 macrophage activation via the Syk-NF-κB mechanism in vivo and in vitro. **A** Western blot detected the protein levels of p-p65, p65, p-Syk and Syk in skin of each group, *n* = 6 in each group. **B** Western blot results showed the protein levels of p-p65, p65, p-Syk and Syk in skin of each group, *n* = 4 in each group. **C** Western blot results showed the protein levels of p-p65, p65, p-Syk and Syk in skin of WT and Mincle-KO mice, *n* = 4 in each group. **D** Identification of the differentiation of mouse bone marrow-derived macrophages (BMDM) by immunofluorescence. **E**–**H** Western blot results of Mincle, iNOS and MyD88 protein levels in BMDM cells, *n* = 3 in each group. **I**–**K** Western blot results of p-p65, p65, p-Syk and Syk protein levels in BMDM cells, *n* = 3 in each group. **L** The results of Real-time PCR showed that expression levels of Mincle, iNOS, IL-1β and TNF-α in BMDM cells, *n* = 3 in each group. **M** ELISA analyzed the secretion of IL-1β, IL-6 and TNF-α in the supernatant of BMDM cells, *n* = 3 in each group. ^@@^*P* < 0.01, ^@@@^*P* < 0.001 vs. Ctrl mice; ^%^*P* < 0.05 vs. IMQ mice; ^###^*P* < 0.001 vs. IMQ + Lipo group; ^&&&^*P* < 0.001 vs. IMQ + Lipo+BMDM group; ^▲▲▲^*P* < 0.001 vs. WT-IMQ mice; **P < 0.01, ***P < 0.001 vs. LPS group.

We next investigated the possible mechanism through which Mincle induces M1 macrophages to mediate psoriasis in vivo and in vitro. Consistent with the previous notion that Mincle signals through the Syk-NF-κB pathway to mediate acute kidney injury [22], we also found that Syk and NF-κB signaling was highly activated in the skin lesions of psoriasis (Fig. 5A). Deletion of Mincle from macrophages significantly inhibited the activation of Syk and NF-κB/p65, suggesting that activation of the Mincle-Syk-NF-κB pathway contributes to macrophage-mediated inflammatory responses in psoriasis in vivo (Fig. 5B, C). This was further confirmed in vitro as knockdown of Mincle from BMDMs blocked LPS-induced activation of Syk-NF-κB pathway, thereby inhibiting M1 macrophage activation and expression of proinflammatory cytokines. The immunofluorescence results showed that the all of extracted primary macrophages expressed F4/80, indicating that the bone marrow cells successfully induced to macrophages (Fig. 5D). The western blot results showed that under the stimulation of LPS, the protein levels of Mincle, iNOS and MyD88 increased significantly, and these three indicators were strongly reduced after Mincle was knocked down (Fig. 5E–H). Similarly, after knocking down Mincle in macrophage, the activities of Syk/NF-κB was significantly reduced (Fig. 5I–K). In addition, after Mincle knockdown, the mRNA expression and secretion levels of inflammatory factors are also significantly reduced (Fig. 5L, M). These results indicate that Mincle regulates Syk/NF-κB pathway to activate the inflammatory response of macrophage.

### Mincle mediates macrophage-dependent skin epithelial cell injury via a PU.1 mechanism in vitro

**Fig. 6:**
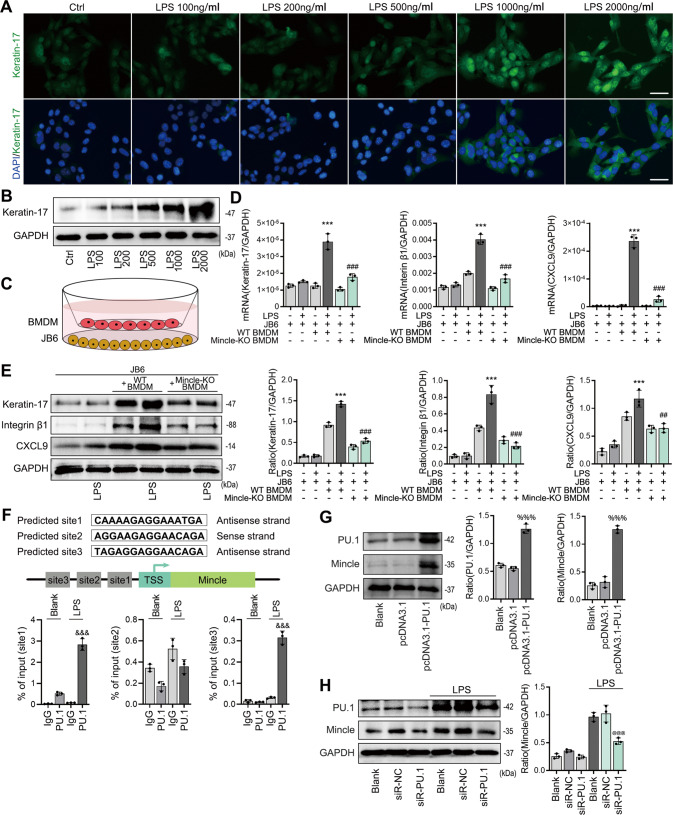
Mincle mediates macrophage-dependent skin epithelial cell injury via a PU.1 mechanism in vitro. **A** Representative images of immunofluorescence staining of keratin-17 in LPS-stimulated JB6 cells. **B** Western blot showed the keratin-17 protein level in LPS-stimulated JB6 cells. **C** The co-culture system of BMDM and JB6 cells. **D** Real-time PCR detected the mRNA expression of Keratin-17, Intergrin β1 and CXCL9 in JB6 co-cultured with or without BMDM. **E** Western blot detected the protein levels of Keratin-17, Intergrin β1 and CXCL9 in JB6 co-cultured with or without BMDM. **F** The predicted binding sites of PU.1 on promotor of Mincle, and the ChIP results demonstrated the PU.1 bingding ability of each site on Mincle promoter. **G** Western blot results showed the protein levels of PU.1 and Mincle after overexpression of Mincle in BMDM cells. **H** Western blot results showed the protein levels of PU.1 and Mincle after knockdown of PU.1 in ctrl and LPS stimulated cells. *n* = 3 in each group. ***P < 0.001 vs. JB6 + LPS group; ^#^*P* < 0.05, ^###^*P* < 0.001 vs. JB6 + BMDM + LPS group; ^%%%^*P* < 0.001 vs. pcDNA3.1 group; ^@@@^*P* < 0.001 vs. LPS group.

We further investigated the potential mechanism of Mincle in macrophage-mediated epidermal cell damage by co-culturing mouse epidermal cells JB6 with BMDM isolated from Mincle^flox/flox^/Lyzcre ^+/+^ or Mincle^flox/flox^/Lyzcre^−/−^ mice under presence or absence of LPS. We found that addition of LPS dose-dependently induced epidermal cell injury by increasing expression of keratin-17 in JB6 cells, with doses ≥ 1000 ng/ml as determined (Fig. 6A, B). Thus, LPS at 500 ng/ml was used in a cell co-culture model (Fig. 6C). Interestingly, the mRNA and protein levels of the epidermal cell damage-related molecules keratin-17, integrin β1 and CXCL9 in JB6 cells were not increased in LPS (500 ng/ml)-stimulated JB6 cells, but became significantly upregulated when co-cultured with Mincle-expressing BMDMs but not with Mincle-KO BMDMs (Fig. 6D, E), demonstrating a Mincle-dependent macrophage-mediated epidermal cell injury in vitro. Importantly, we also found that LPS-induced upregulation of Mincle on BMDMs was associated with a marked expression of PU.1. Further analysis showed that PU.1 has 3 potential binding sites in the Mincle promoter region (Fig. 6F). ChIP analysis revealed that PU.1 could bind to the target 1 site in the Mincle promoter region, which was increased under the stimulation of LPS (Fig. 6F). Functionally, we also found that overexpression of PU.1 in macrophage significantly increased the protein level of Mincle, which was reversed by knockdown PU.1 (Fig. 6G, H), indicating that PU.1 transcriptionally regulates the expression of Mincle in macrophage. These results suggest that PU.1 may be a key transcriptional factor in regulating Mincle expression and macrophage-mediated psoriasis.

### Targeting Mincle improves the skin lesion in psoriasis mouse model

**Fig. 7:**
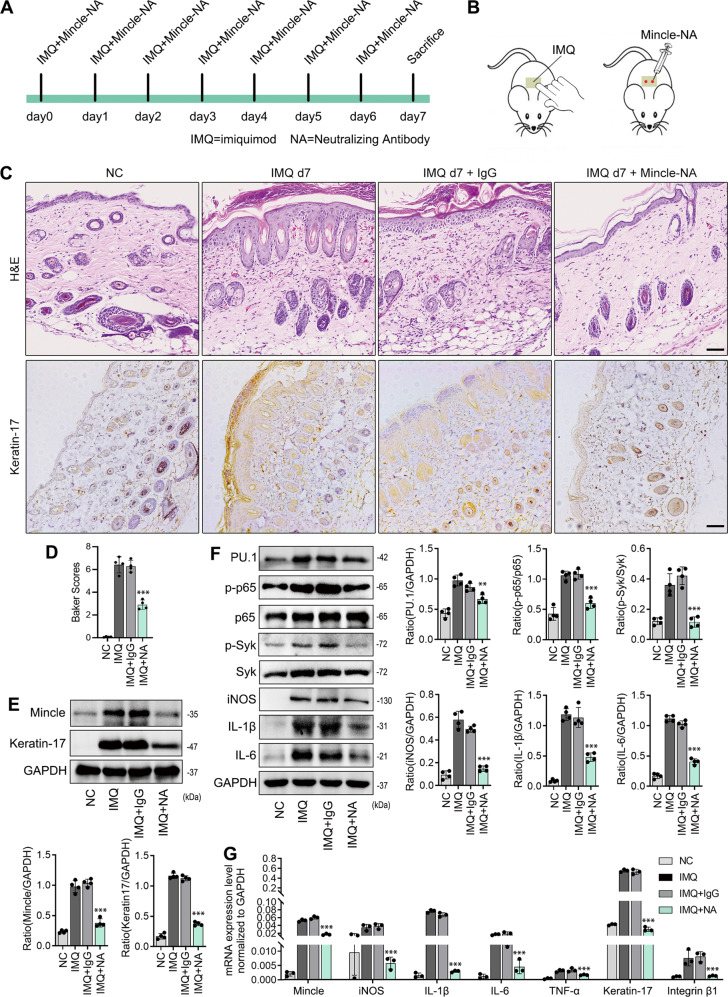
Targeting Mincle improves the skin lesion in psoriasis mouse model. **A** Timeline of subcutaneous injection of Mincle neutralizing antibody in psoriasis mice. **B** Schematic diagram of administration of Mincle neutralizing antibody to mice. **C** H&E and immunohistochemistry staining showed the histopathology and expression of keratin-17 of skin in Ctrl and Mincle neutralizing antibody-treated psoriasis mice. **D** Baker score of H&E staining. **E** Western blot detected the protein levels of Mincle and keratin-17 in each group. **F** Western blot results showed the level of inflammation-related proteins including PU.1, p-p65, p65, p-Syk, Syk, iNOS, IL-1β and IL-6 in each group. **G** Real-time PCR analyzed the mRNA expression of Mincle, iNOS, IL-1β, IL-6, TNF-α, keratin-17 and intergrin β1 in each group. *n* = 4 in each group. **P < 0.01, ***P < 0.001 vs. IMQ group.

We next developed a therapeutic potential for psoriasis by treating psoriasis mice with a Mincle neutralizing antibody subcutaneously for 7 consecutive days (Fig. 7A, B). Results showed that treatment with a Mincle neutralizing antibody significantly improved the psoriasis-like skin damage induced by imiquimod by inhibiting Mincle-expressing macrophage infiltration and blocking Syk/NF-κB-driven expression of iNOS, IL-1β, IL-6, TNF-α, and keratin-17 and integrin β1 expression in the psoriasis skin (Fig. 7C–G). Interestingly, blockade of Mincle also inhibited PU.1 expression (Fig. 7F), indicating that there is a feedback loop between PU.1 and Mincle.

A simplified pathway model of Mincle in macrophage-mediated psoriasis is illustrated in the graphical abstract.

## Discussion

It is well established that macrophages play a key role in the pathogenesis of psoriasis as depletion of macrophages have been shown to inhibit the severity of skin lesions in mouse models of psoriasis [14]. Consistent with this finding, we also found that there were a marked M1 macrophages identified by co-expressing CD68(F4/80) and Mincle (a receptor for DAMPs and PAMPs on macrophages) in the skin lesions of patients and IMQ-induced animal model of psoriasis and that depletion of macrophages locally in the psoriasis-like skin lesions with clodronate liposomes inhibited the severity of skin injury. Importantly, our present study discovered that macrophages mediated psoriasis via the Mincle-dependent mechanism. This was supported by the findings that adoptive transfer with Mincle-expressing macrophages but not Mincle-KO macrophages largely promoted psoriasis-like lesions. Furthermore, by using macrophage-specific Mincle KO mice (Mincle^loxp/loxp^/Lyz2-cre^+/+^), we also found that specific deletion of macrophage Mincle produced a protective effect on M1 macrophage-mediated psoriasis. Thus, Mincle plays a critical role in macrophage-mediated psoriasis in a mouse model of IMQ-induced psoriasis.

Psoriasis is the most common inflammatory skin disease [23], and its incidence has increased significantly in the past two decades [24]. It is currently believed that psoriasis is a multifactorial disease, which mainly occurs in individuals with genetic susceptibility, leading to a series of complex inflammatory cascades [25]. Current researches reported that this disease is triggered and maintained by the interaction of innate and adaptive immune cells, such as dendritic cells, T cells, and neutrophils [26]. Singh et al. found that psoriasis inflammation requires DCs or their precursors to express CCR6, which mediates the transport of monocytes to the inflamed skin, and then produces inflammatory cytokines to enhance the activation of skin T cells, which is the key factor leading to psoriasis [27]. Another report also indicated that specific chemokines may recruit memory T cell subpopulations into the skin, and these T cells may be activated and expanded by DCs to maintain different immune polarities in psoriasis [28]. Herster et al. reported that RNA and antimicrobial peptide LL37 are massively complexed in neutrophil extracellular trap (NETs) in skin of psoriasis, triggering the release of TLR8/TLR13-mediated cytokines and NETs in neutrophils in vitro and in vivo, which further promotes inflammation [29]. However, a large number of macrophages also infiltrate to the psoriasis lesions [30, 31], and its role in psoriasis needs further study. A recent study has reported that subcutaneous injection of clodronate liposomes eliminates skin macrophages, resulting in a significant reduction in psoriasis-like skin changes, suggesting the important role of macrophages in psoriasis injury [14]. This result is consistent with the results obtained by deleting skin macrophages at the lesion site in our experiments, and further cell experiments also confirmed that the cross-talk between macrophages and epidermal cells aggravated the damage of epidermal cells.

Macrophage polarization plays a key regulatory role in the inflammatory response of macrophages [32, 33]. In different environments, macrophage can differentiate into two types of cells with opposite functions. Under the stimulation of inflammatory condition such as interferon and TNF-α, macrophage can differentiate into M1-type macrophage, which secretes a large amount of IL-1β, IL-6, TNF-α, iNOS, CCL2, CCL3, CXCL9 and other inflammatory factors, chemokines as well as their ligands, further promoting inflammatory response [34]. However, the M2-type macrophage can secrete antagonists of inflammatory factors and matrix metalloproteinases, which are mainly anti-inflammatory [35]. Mincle, a pattern recognition receptor, has been reported to maintain the M1 phenotype of macrophage and promot inflammation in multiple diseases, such as acute kidney injury [22], hemorrhage [36], obesity [37] and cancer [38], but its role in psoriasis is unclear. In order to answer this question, we tested the expression of Mincle in skin of psoriasis patients and psoriasis-like mouse models induced by miquimod, and found that it was strongly upregulated in both human and mouse psoriatic lesions compared to normal group. It was also found that the M1 macrophage marker iNOS was significantly increased in psoriasis by flow cytometry, and the expression of iNOS was significantly reduced both in vivo and in vitro when Mincle was knocked out, indicating that Mincle maintained the M1 polarization of epidermal macrophage and promoted the inflammatory response and skin damage associated with its downstream signaling Syk and NF-κB in psoriasis. Importantly, we found that the transcription factor PU.1, crucial for the development of macrophages in mammals, may bind to the promoter region of Mincle through bioinformatics analysis. PU.1 is a member of the E26 − transformation−specific (Ets) family of transcription factors [39], which acts as an important factor in macrophage maturation by regulating the expression levels of genes involved in cell differentiation [40]. PU.1 is also involved in macrophage-involved inflammation, studies have shown, PU. 1 activates macrophages and increases the inflammatory response by regulating NLRP3 inflammasome [41]. Interestingly, PU.1 may serve as an important target for vitamin D3 to alleviate atopic dermatitis, a severe inflammatory skin disease [42]. Based on the bioinformatics data, we speculated that PU.1 may be a key transcription factor regulating Mincle and the inflammation maintained by which. In this study, the ChIP results confirmed that PU.1 binds tightly to one of the three predicted binding sites, overexpression and knockdown of PU.1 increased and reduced the expression of Mincle in vitro, indicating that the inflammatory response of macrophages involved in Mincle is related to PU.1 transcriptional regulation.

Since Mincle is believed to promote psoriatic damage, targeting Mincle may be a new way to treat psoriasis. However, no Mincle inhibitor is currently available. In the previous study, we used the inhibitor of Syk, a downstream of Mincle, to interfere with the transmission of Mincle signal and achieved a certain anti-inflammatory effect [43], but its potential off-target effect and inhibition of non-Mincle limit its application in the treatment of psoriasis. For this reason, we used Mincle neutralizing antibody to explore its potential therapeutic effects, and found that Mincle neutralizing antibody can significantly inhibit imiquimod-induced psoriasis-like skin damage, inhibit inflammation, and present a significantly anti-psoriatic effect.

The keratinocyte in healthy skin has an active proliferation capability that secretes the normal differentiation of the marker keratin1 and keratin10, However, in psoriasis, keratinocytes face a variety of inflammatory stimulation leading reduced expression of keratin1 and keratin10, while keratin6, keratin16 and keratin17 are abnormally upregulated [44], which indicates them are the important markers of abnormal keratinocyte keratinization in the process of psoriasis. Thus, we chose keratin17 as the marker of epidermal injury in this study.

In this present study, we detected the expression pattern of Mincle in the skin of psoriasis patients and mouse models, and explored the promotion of Mincle on psoriasis inflammation and damage by knocking out Mincle in macrophage, and used Mincle neutralizing antibody to develop a novel therapeutic target and drug for psoriasis, providing new direction and option for the clinical treatment of disease.

## Materials and methods

### Clinical samples

The skin samples from 10 psoriasis patients were collected from the department of pathology, Affiliated Traditional Chinese Medicine Hospital of Southwest Medical University. 10 of normal skin samples were obtained from non-psoriatic orthopedic surgery. This study was approved by the Ethics Committee of Affiliated Traditional Chinese Medicine Hospital of Southwest Medical University (The approval number: KY2021013).

### Animals

C57BL/6 mice (Male, 22–25 g) used in this study were pruchased from Chongqing Tengxin Biotechnology Co., Ltd. The Mincle^flox/flox^ mice (C57BL/6-Clec4e^tm1.1cyagen^) was constructed by Cyagen Biosciences (Guangzhou, China), and Lyz2-cre mice was also obtained from Cyagen Biosciences. The tdTomato mince (B6.129(Cg)-Gt(ROSA)26Sor^<tm4(ACTB-tdTomato,-EGFP)Luo>^/J) were pruchased from The Jackson Laboratory. The Mincle^flox/flox^ mice were crossed with Lyz2-cre mice to obtain Mincle conditional knock-out mice (Mincle^flox/flox^/Lyz2-cre^+/+^). The grouping of mice was randomized, with 6 mice in each group. All mice were housed in a temperature- and humidity-controlled room with a 12 h light/12 h dark cycle. All animal experiments were performed according to the guidelines from the Ethics Committee of the Southwest Medical University.

### The psoriasis mice model

Mice were received a daily dose of 62.5 mg imiquimod (IMQ) cream on the shaved back of 3.0 cm × 2.0 cm for 7 consecutive days to construct the psoriasis mice model. The control mice were treated similarly with a vehicle cream (Vaseline cream). The blood and skin samples were collected on the 7th day after treatment. Finally, the skin samples were assessed using Baker’s scoring system according to H&E stainning.

### Extraction and reinjection of BMDM

The bone marrow-derived macrophage (BMDM) cells were isolated from WT, Mincle^flox/flox^/Lyz2-cre^+/+^ and tdTomato mice and cultured in DMEM medium with 30% supernatant of L929 cells. Briefly: Carefully separate the hind leg bone from the femur and remove the attached muscles. Subsequently, the bones were washed with sterile PBS and 75% of alcohol for 3 times, respectively. Transfer all the cleaned bones to a sterile mortar, add 5 ml PBS to grind the bones for 30 s, followed by collecting the liquid into a 50 ml centrifuge tube, and repeat the operation for 4 times. All the liquid were treated with Red Blood Cell Lysis Buffer to break the erythrocytes and then filtered on a 40 μm cell strainer. After centrifuging the collected liquid at 1500 rpm for 5 min, the obtained cells were seeded in a 10 cm dish with complete medium (DMEM with 10% FBS, 30% L929 supernatant, 100 U/ml penicillin and 100 mg/ml streptomycin). BMDM cells can be harvested 7 days later.

In BMDM reinjection experiment, mice were reinjected with 50 μL of suspension containing 5 × 10^6^ BMDM cells through the tail vein.

### Cell culture

Since the differentiation of macrophage needs the macrophage colony factor, the BMDM cells were culture in DMEM medium containing 10% FBS and 30% supernatant of L929 cells. The L929 cell line was purchased from Shanghai Cell Bank of Chinese Academy of Sciences, and cultured in 1640 with 10% FBS. Collecting the supernatant of L929 cells at the day 5 post seeding in a 10 cm dish. The JB6 cell line was obtained from Ningbo University School of Medicine and cultured in RPMI 1640 with 10% FBS. All medium to culture each kind of cells containing 1% penicillin-streptomycin, and under an incubative condiction of 37 °C and 5% CO_2_.

### H&E staining

Fresh skin tissues were fixed in PBS-prepared 10% formalin solution for 24–48 h. Subsequently, the tissues were sequentially placed into a concentration gradient ethanol solution for dehydration, followed by two transparent treatments in xylene for 30 min each. Then, samples were embedded in paraffin, and cut into 4 μm serial sections. After dewaxing and rehydration, the sections were stained with hematoxylin-eosin (HE) reagent to identify skin tissue structures and cell nuclei, dried and sealed with neutral resin. Images were captured by a light microscope (DM500, Leica, Germany).

### Immunohistochemical staining

The tissue sections were washed with PBS for two times, followed by incubating samples in sodium citrate buffer (pH = 6.0) for heat-mediated antigen repair with 3% hydrogen peroxide at room temperature. After washing with PBS for two times, samples were blocked with 5% BSA for 30 mins at room temperature. Then, samples were incubated with primary anti-human Mincle, IL-6, TNFα and anti-mouse keratin-17 antibody (1:200) overnight at 4 °C, and following incubated with biotin-labeled secondary antibody working solution for 1 h at room temperature. Ultimately, the positive staining was visualized by using DAB kitt. Images were captured by a light microscope (DM500, Leica, Germany). The IOD values were calculated using Image Pro Plus 6.0 software.

### Immunofluorescence staining

Skin tissues were fixed with 10% formalin for 24 h, followed by dehydrating in 10% sucrose for 1 h, 20% sucrose for 5 h, and 30% sucrose overnight. The tissues were cryo-embedded in OCT with liquid nitrogen, cut into 4 μm serial sections. The sections were washed 3 times with PBS, blocked at room temperature for 30 min (5% BSA in PBS), followed by incubating with primary antibody (1:100) overnight at 4 °C. Then, samples were incubated with fluorescent secondary antibody (1:200) for 1 h at room temperature, and stained with DAPI to visualize the nucleus. The images were captured by EVOS system.

### Real-time PCR

Total RNA from skin and BMDM cells were isolated by using TRIzol Reagent (TianGen, DP419, China). The reverse transcription was performed to obtain cDNA by using the HiScript III RT SuperMix (Vazyme, R323-01, China). Finlly, quantitative PCR was performed using ChamQ Universal SYBR qPCR Master Mix (Vazyme, Q711-02, China) on the LightCycler® 480 II Real-Time PCR System (Roche, Germany). The primers were synthesized by Sangon Biotech (Shanghai, China), and the sequences are shown in Table 1.

**Table 1:** Specific primers for real-time PCR.

Gene	Primer sequence (5’ to 3’)
Mincle	S: ACCAAATCGCCTGCATCC
	A: CACTTGGGAGTTTTTGAAGCATC
Keratin17	S: ACCATCCGCCAGTTTACCTC
	A: CTACCCAGGCCACTAGCTGA
Integrin β1	S: CGTGGTTGCCGGAATTGTTC
	A: ACCAGCTTTACGTCCATAGTTTG
CXCL9	S: TCCTTTTGGGCATCATCTTCC
	A: TTTGTAGTGGATCGTGCCTCG
IL-1β	S: TGCCACCTTTTGACAGTGATG
	A: AAGGTCCACGGGAAAGACAC
IL-6	S: AAAGAGTTGTGCAATGGCAATTCT
	A: AAGTGCATCATCGTTGTTCATACA
TNF-α	S: CATCTTCTCAAAATTCGAGTGACAA
	A: TGGGAGTAGACAAGGTACAACCC
iNOS	S: CAGCTGGGTCGTACAAAC
	A: CATTGGAAGTGAAGCGTTT
GAPDH	S: ACAGCAACAGGGTGGTGGAC
	A: TTTGAGGGTGCAGCGAACTT

### Western blotting

Protein of skin tissue were extracted from 50 mg of mouse epidermal tissue by liquid nitrogen grinding method. The protein concentrations were determined by the Kormas Brilliant Blue method. 50 μg of protein was subjected to SDS-PAGE electrophoresis, transferred through PVDF membrane, and then locked with 5% skimmed milk at room temperature for 1 h. Mincle (sc-390806, Santa cruz), p-Sky (2717 S, Cell Signaling Technology), Sky (13198 S, Cell Signaling Technology), p-P65 (3033 S, Cell Signaling Technology), P65 (8242 S, Cell Signaling Technology), iNOS (13120 S, Cell Signaling Technology), F4/80 (sc-52664, Santa cruz), IL-1β (sc-52012, Santa cruz), IL-6 (sc-32296, Santa cruz), TNFα (sc-52746, Santa cruz), PU.1 (2258 S, Cell Signaling Technology), keratin-17 (17516-1-AP, Proteintech), Integrin β1 (bs-0486R, Bioss), CXCL9 (bs-2551R, Bioss) primary antibody (dilution ratio 1:1,000) were added and incubated overnight at 4 °C. After washing with TBST for 5 min three times, horseradish peroxidase (HRP) coupled secondary antibody (dilution ratio 1:10000) was added and incubated at room temperature for 1 h. After washing with TBST for 5 min three times, the results were detected by gel imager and expressed as the relative grayscale values of target proteins and internal reference.

### Flow cytometry

1 cm^2^ of skin collected from each group was digested with 0.2% type 4 collagenase for 1 h at 37 °C with 180 rpm shaking. After filtering the supernatant with a 40 μm cell strainer, the obtained single cells were washed with PBS for 3 times. Subsequently, cells were fixed by 4% paraformaldehyde at room tempreture for 15 min, followed by blocking with 5% BSA for 30 min. Next, cells were incubated with 1% BSA diluted primary antibody (Mincle (dilution ratio 1:100), F4/80 (dilution ratio 1:100)) at 4 °C overnight. After washing the cells with PBS for 3 times, cells were incubated with FITC or PE conjugated fluorescent secondary antibody (dilution ratio 1:200) at room tempreture for 1 h. All cells were gated and analyzed by using a flow cytometer (BD bioscience), and the results were analyzed by using Flow Jo software.

### Co-culture system

The BMDM and JB6 cells were co-cultured by using Transwell chamber (Millicell, PIHT30R48, USA) in a 6-well culture plate. Briefly, 2 × 10^5^ of BMDM cells were seeded in a transwell chamber as the upper of the co-culture system, and 5 × 10^5^ of JB6 cells were seeded in a well of 6-well plate as the lower of the system. Add 3 ml of 1640 medium to the co-culture system to make the medium level just over BMDM cells in the upper chamber.

### ChIP

Formaldehyde was quickly added to the culture medium to make the final concentration of formaldehyde to 1%. The cells were then incubated on a shaker to fix the chromatin cross-linking at room temperature for 10 min, and glycine was added to terminate the cross-linking after fixation was completed. Subsequently, the cells were incubated in cell membrane lysis solution to remove the cell membranes, followed by using 10 U/μl DNA restriction endonuclease to cleave the chromatin into 150–1000 bp small fragments. Then, PU.1 antibody was added and incubated overnight. The target chromatin fragments were collected after removing the free chromatin fragments by magnetic bead. In this study, three different primer pairs were designed according to the promoter sequence of Mincle, and finally, the changes of Mincle expression levels before and after DNA enrichment were compared by real-time PCR assay.

### Macrophage deletion and Neutralizing antibody injection

For macrophage deletion: On the second day of IMQ treatment, 200 μl of Clodronate Liposomes (Liposoma) and control liposomes were subcutaneously injected to the IMQ treated skin, respectively.

For neutralizing antibody injection: 5 μg of anti-mMincle-IgG neutralizing monoclonal antibody (mabg-mmcl, Invivogen, USA) or IgG control were subcutaneously injected to the left and right sides of IMQ treated skin, respectively, for 7 days.

### Statistical analysis

Data were processed and statistically analyzed by using SPSS software 21.0 (SPSS, Chicago, IL, USA), and data were expressed as mean ± standard deviation. Student’s *t*-test was used to analyze data between two independent groups, and one-way ANOVA was used to compare data between multiple groups. *P* < 0.05 was considered a statistically significant difference.

## Supplementary information

Supplementary file

WB RAW DATA

## Data Availability

The datasets are available from the corresponding author on reasonable request. Supplementary information is available at Cell Death Discovery’s website.
