# Supplementary material for: Macrophages mediate psoriasis via Mincle-dependent mechanism in mice
Source: Cell Death Discov. 2023 Apr 28;9:140. doi: 10.1038/s41420-023-01444-8 (PMC10147944; doi:10.1038/s41420-023-01444-8)
Supplement: Supplementary file 1 — Supplementary file [file 41420_2023_1444_MOESM1_ESM.docx]

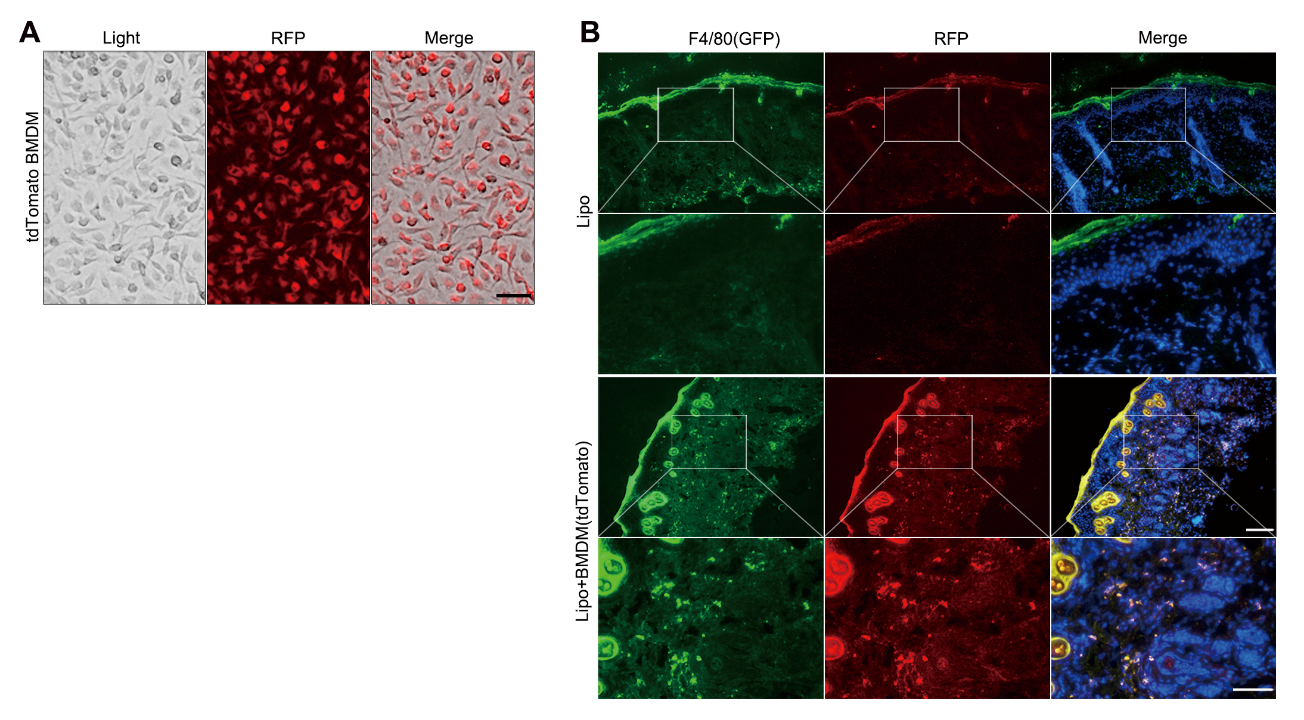


Supplementary Fig 1. (A) The td-tomato(RFP) expression in BMDM; (B) Representative images of immunofluorescence staining of F4/80(GFP) and td-tomato(RFP) in each group.
