# Supplementary figures and images for: Macrophages mediate psoriasis via Mincle-dependent mechanism in mice
Source: Cell Death Discov. 2023 Apr 28;9:140. doi: 10.1038/s41420-023-01444-8 (PMC10147944; doi:10.1038/s41420-023-01444-8)

Fig2-I:

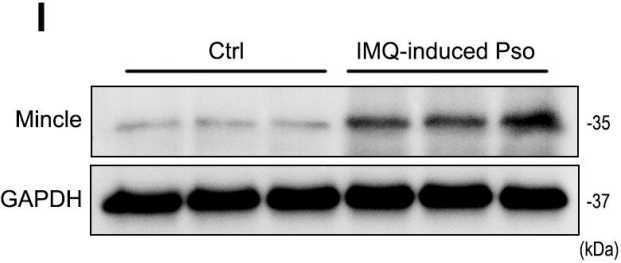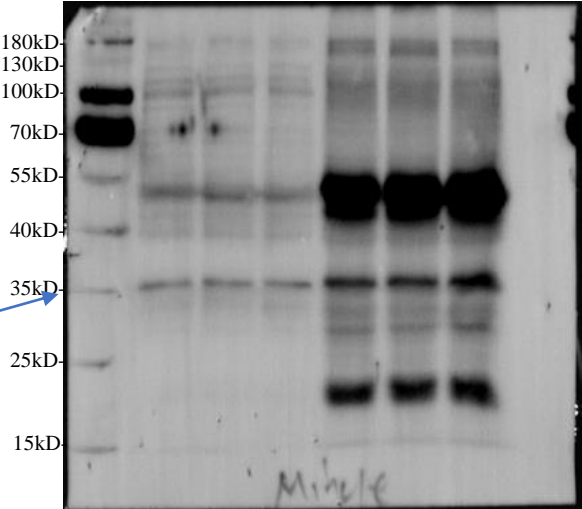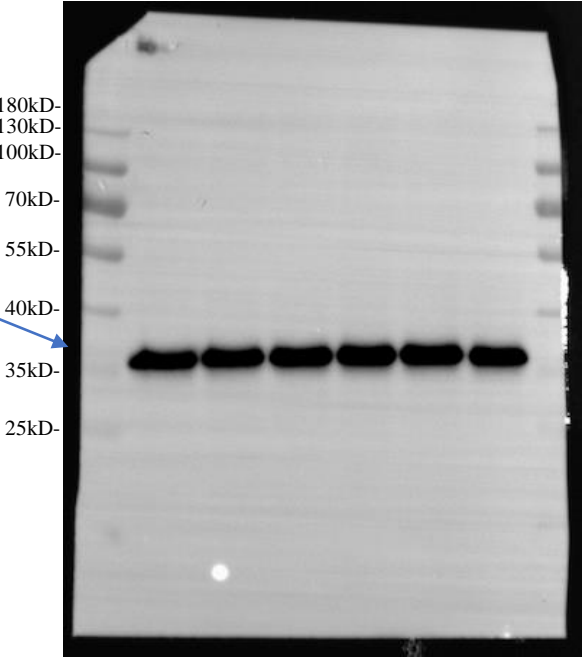

Fig2-K:

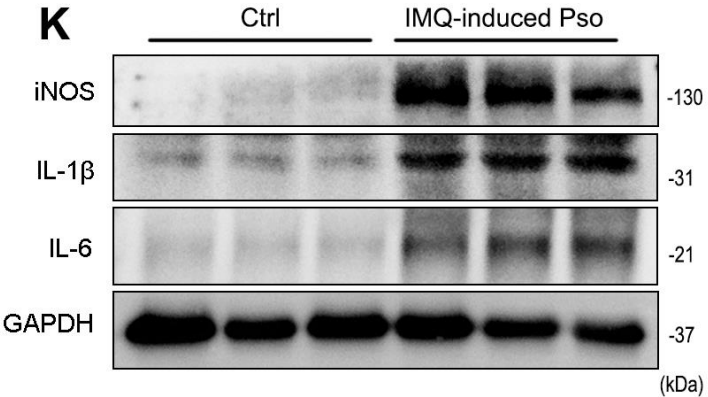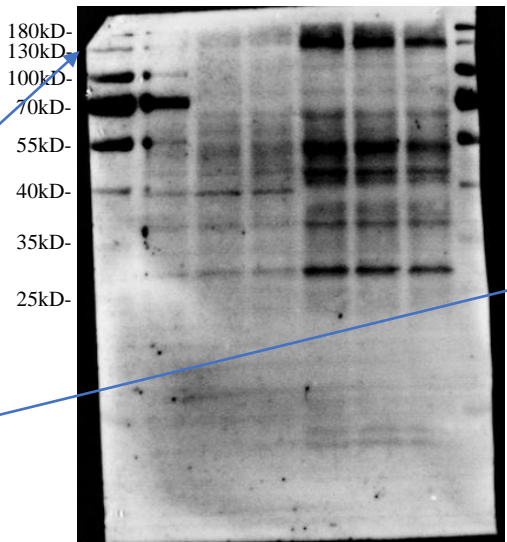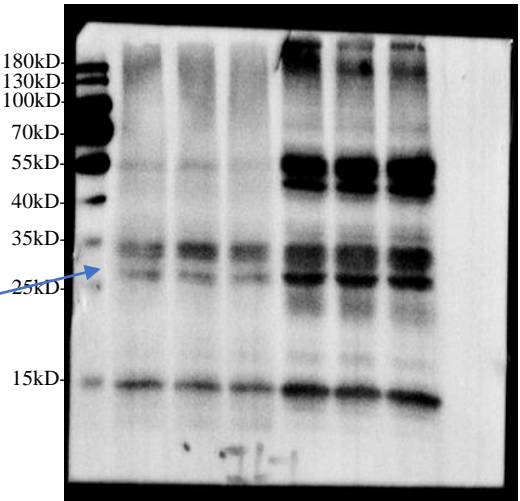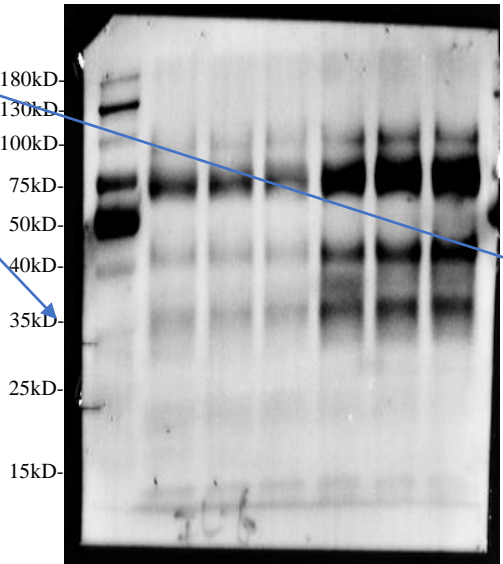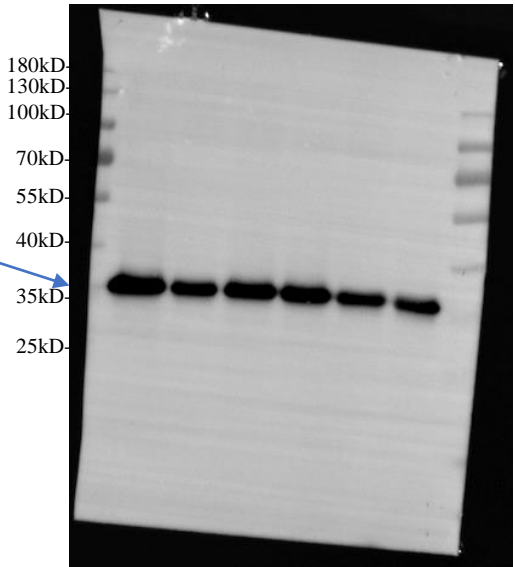

Fig3-G:

G

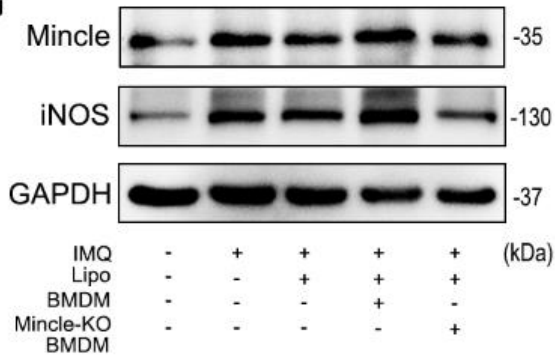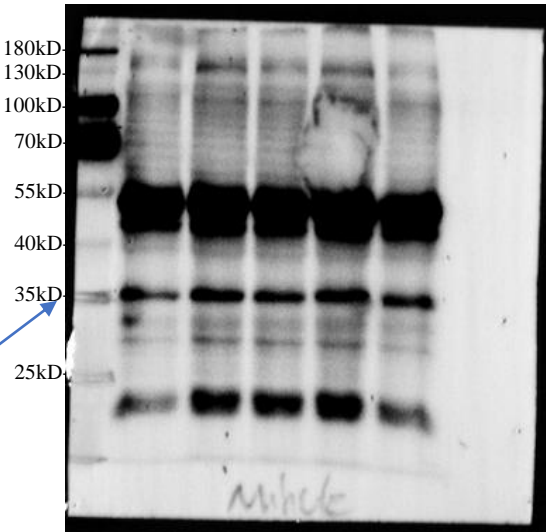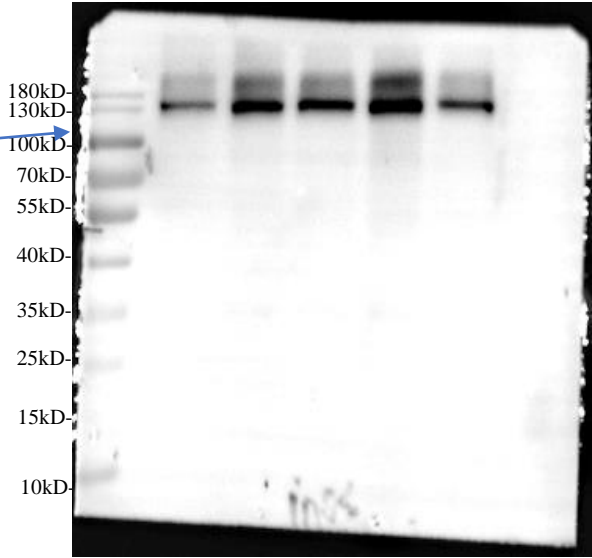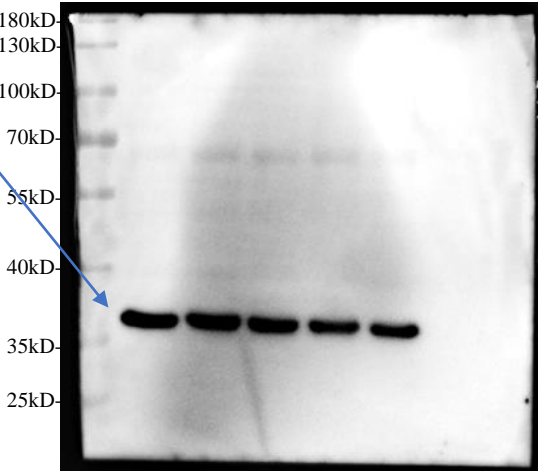

Fig4-E:

**E**

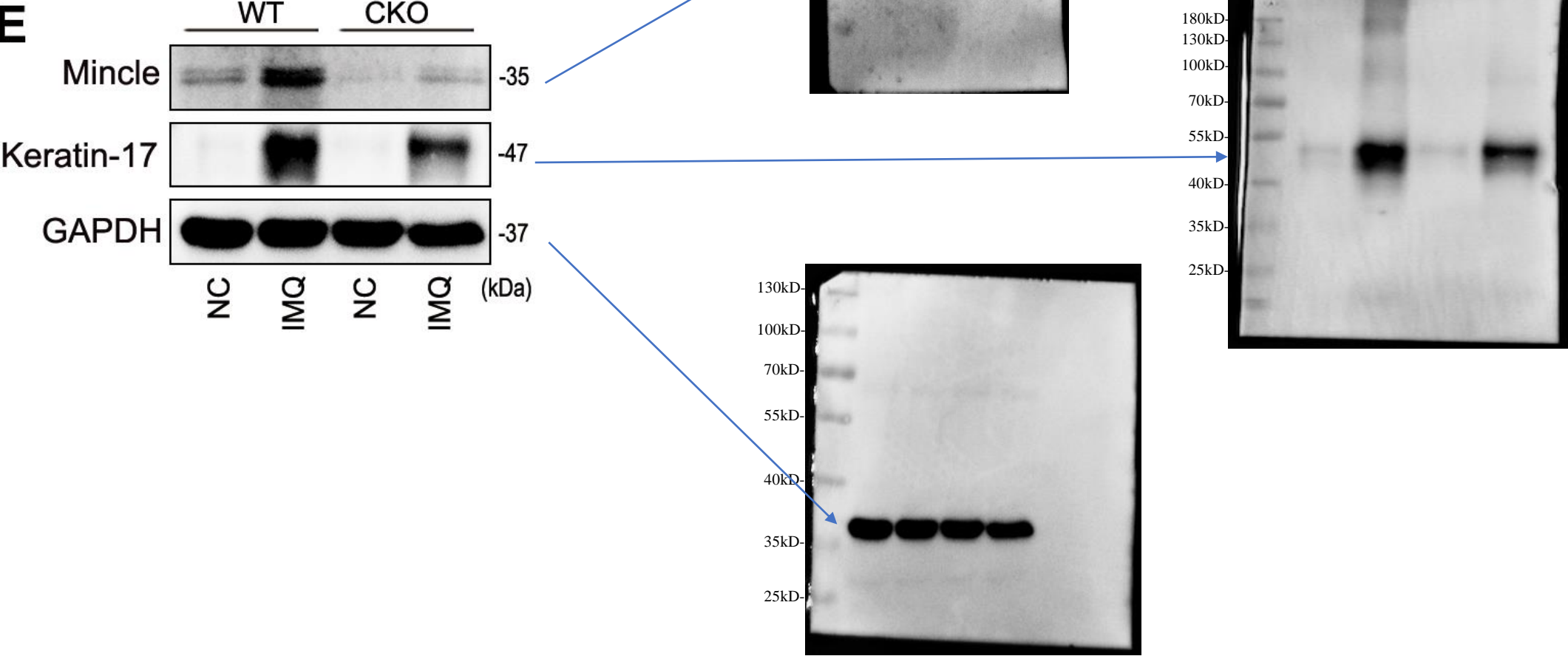

Fig4-F:

**F**

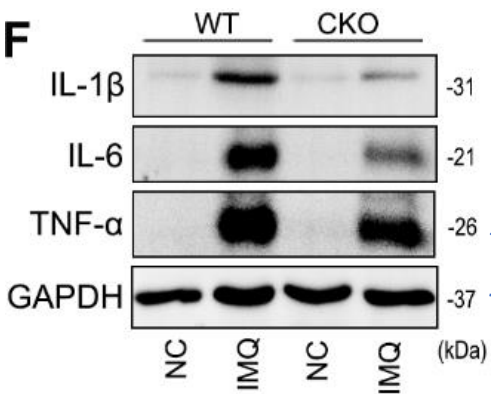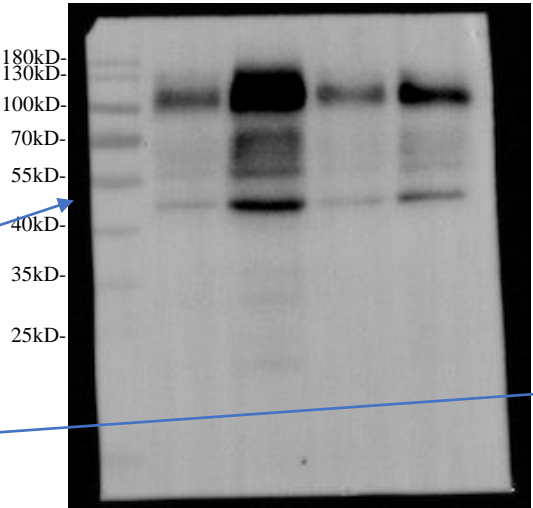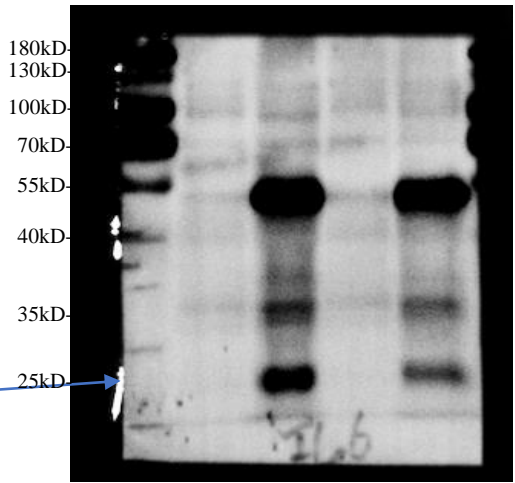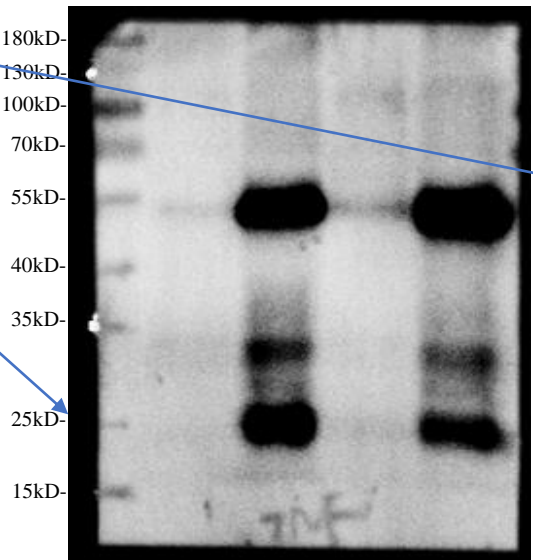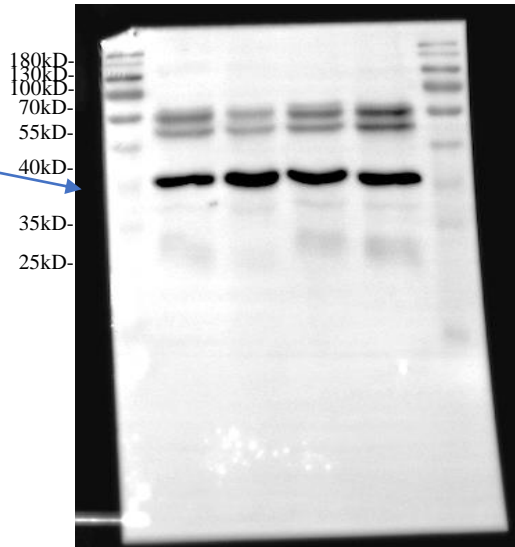



Fig5-B:

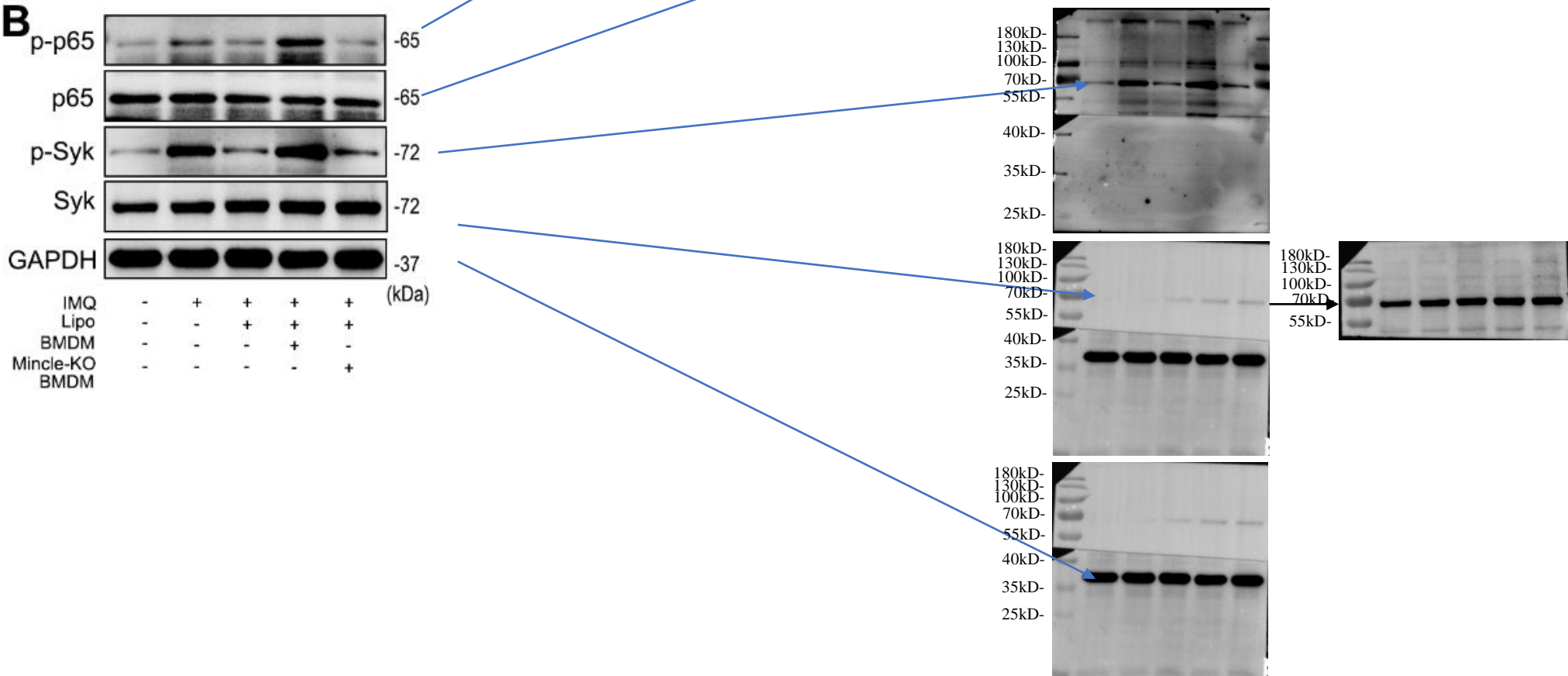

Fig5-C:

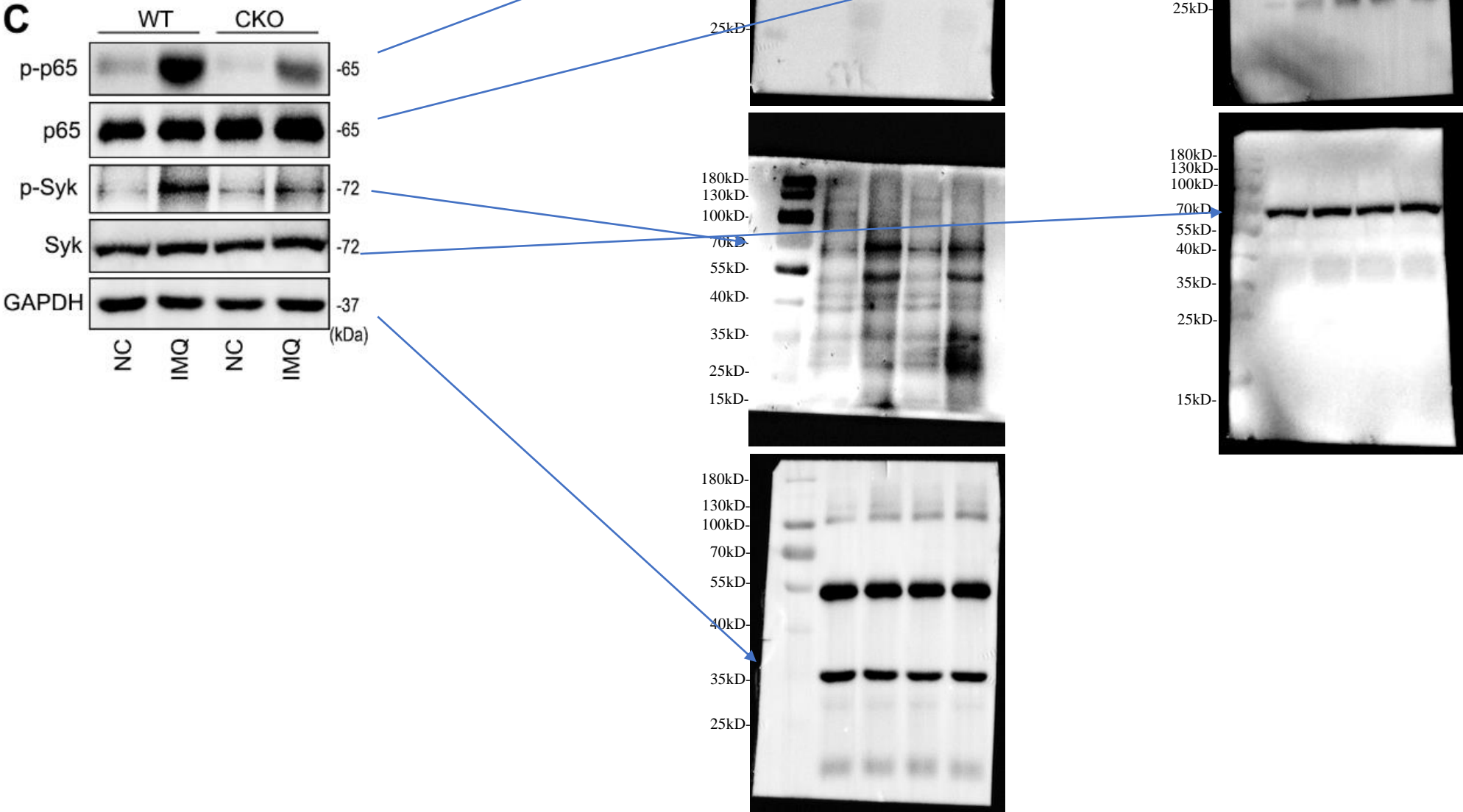

Fig5-E:

E

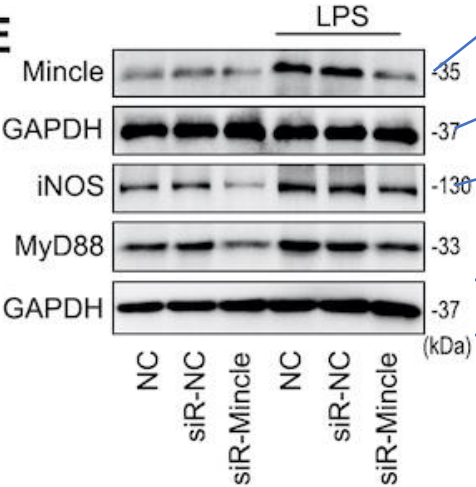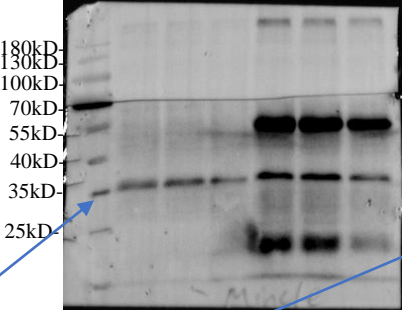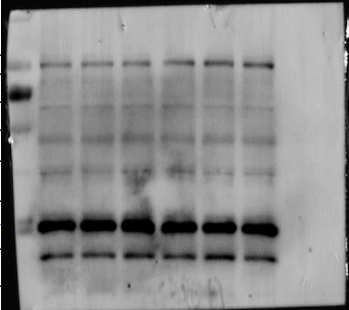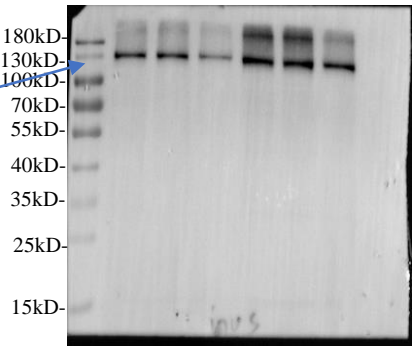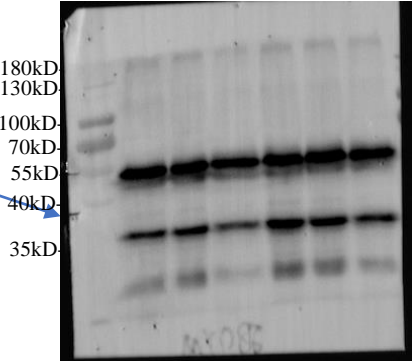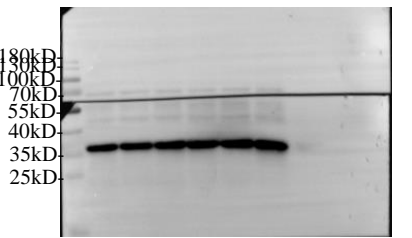

Fig5-I:

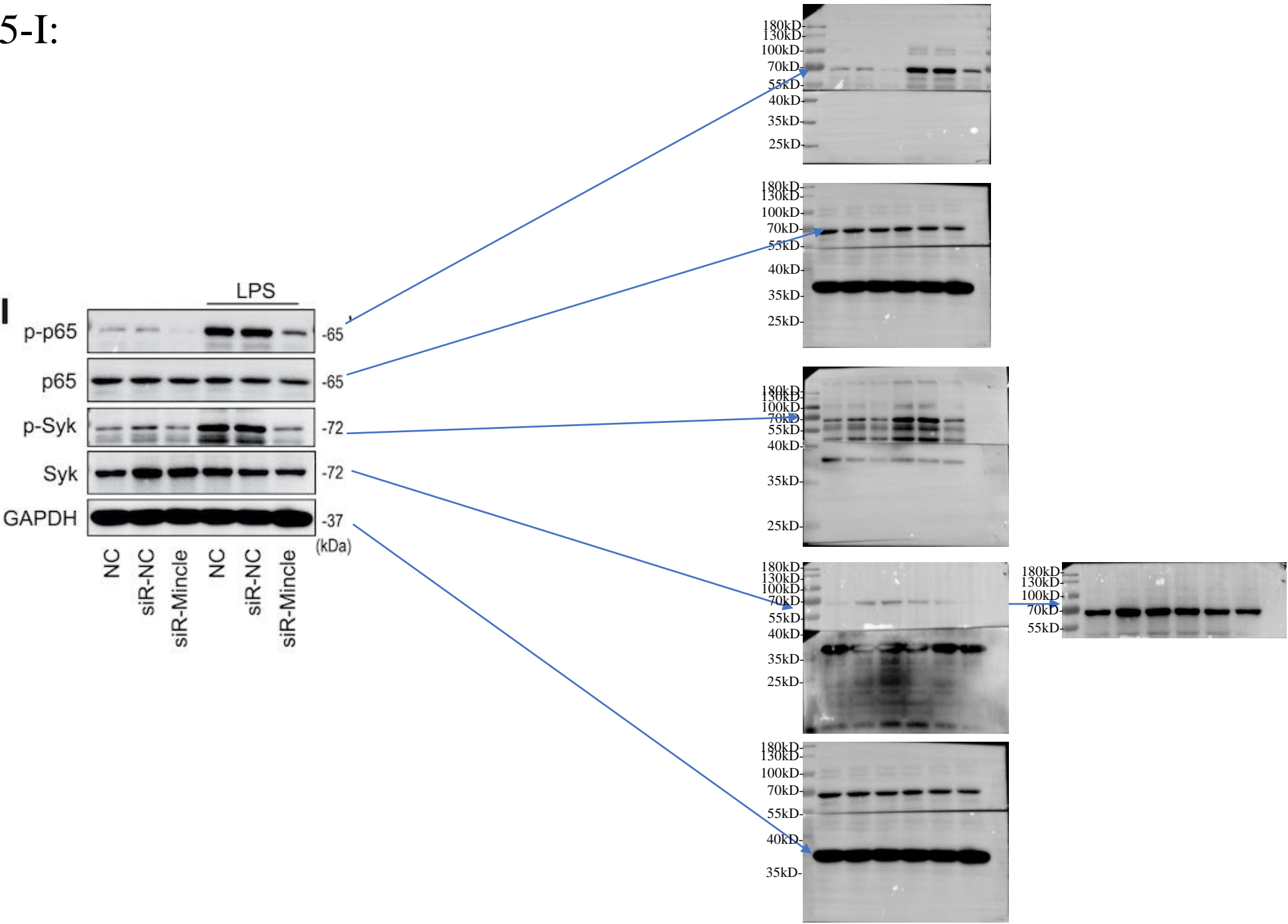

Fig6-B:

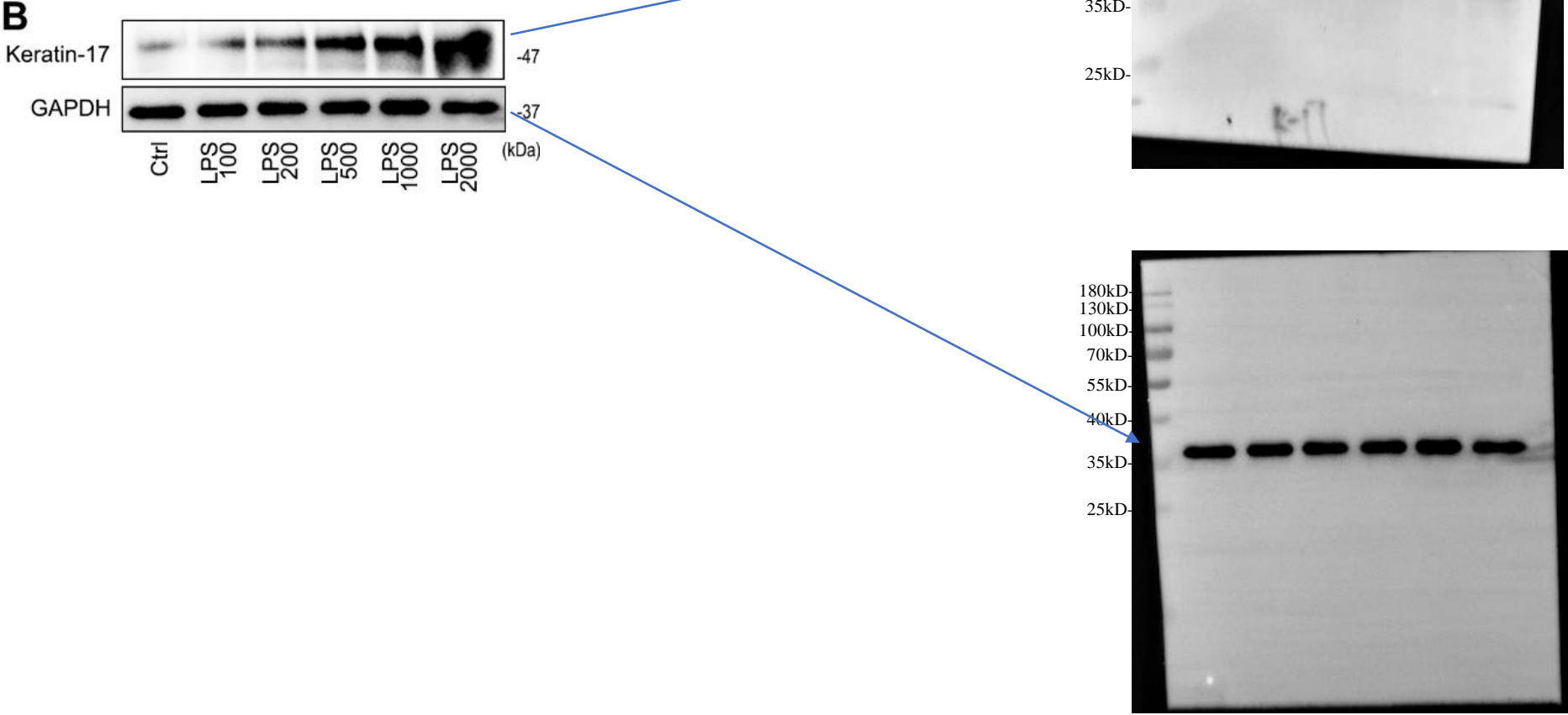

Fig6-E:

E

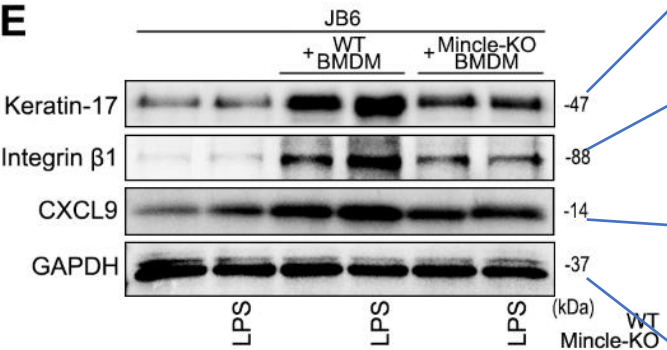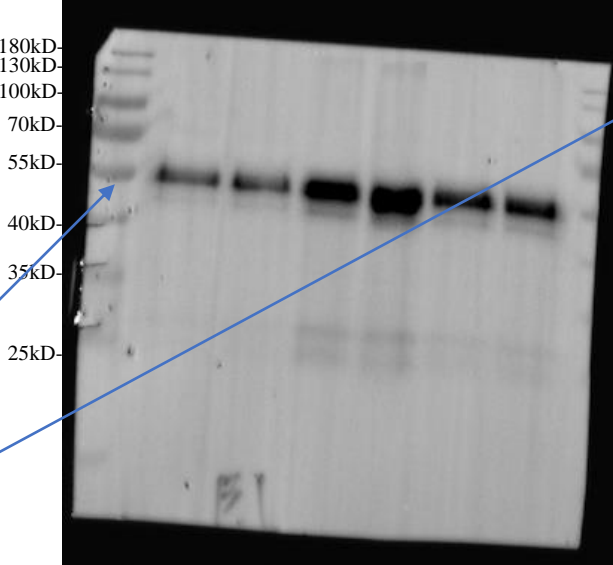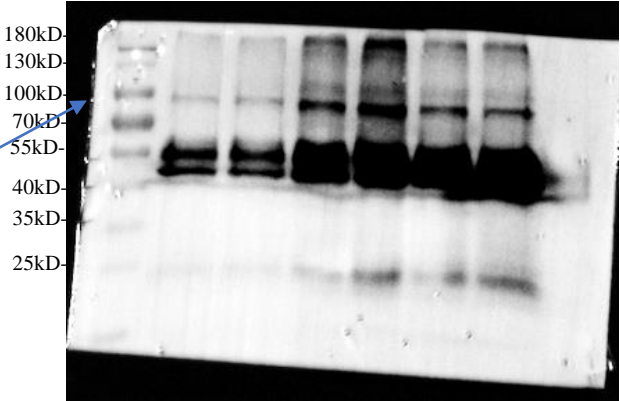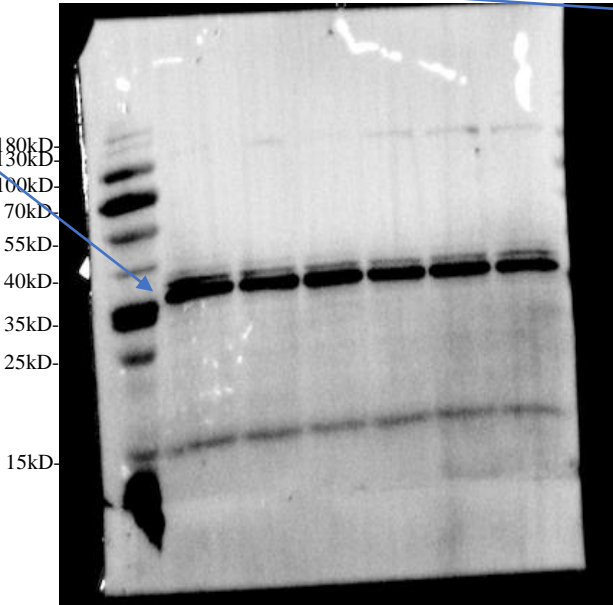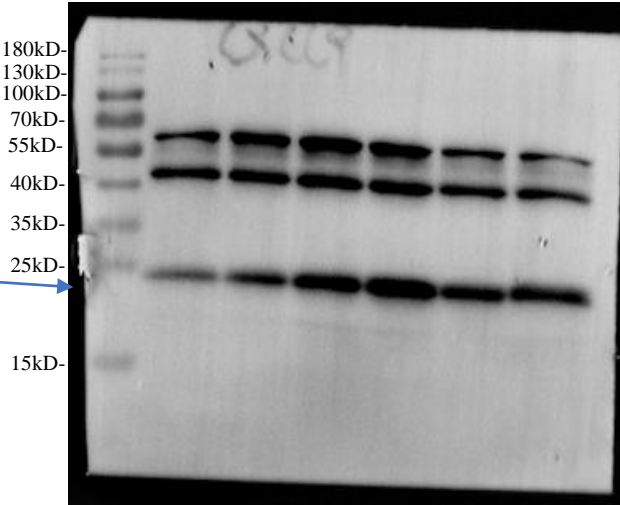

Fig6-G:

**G**

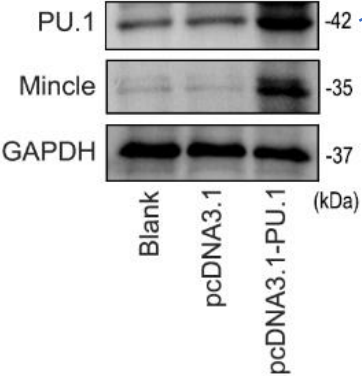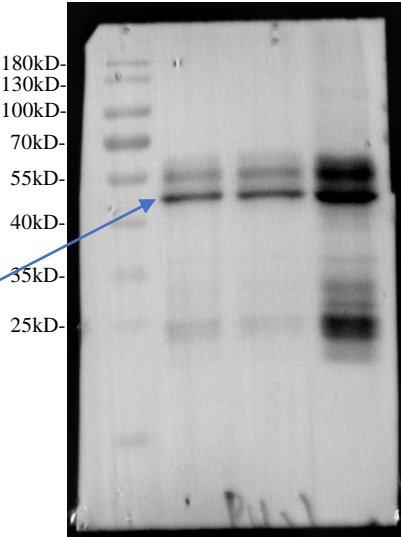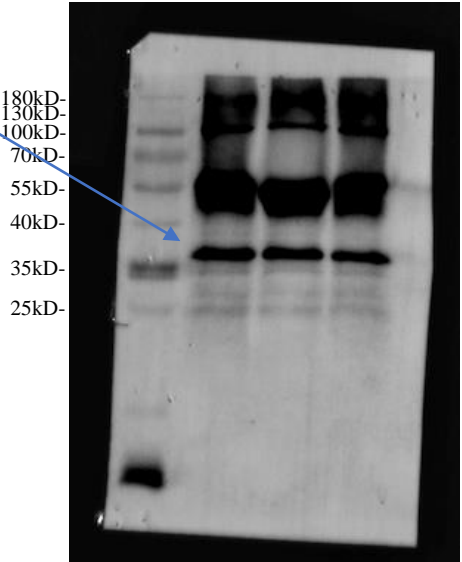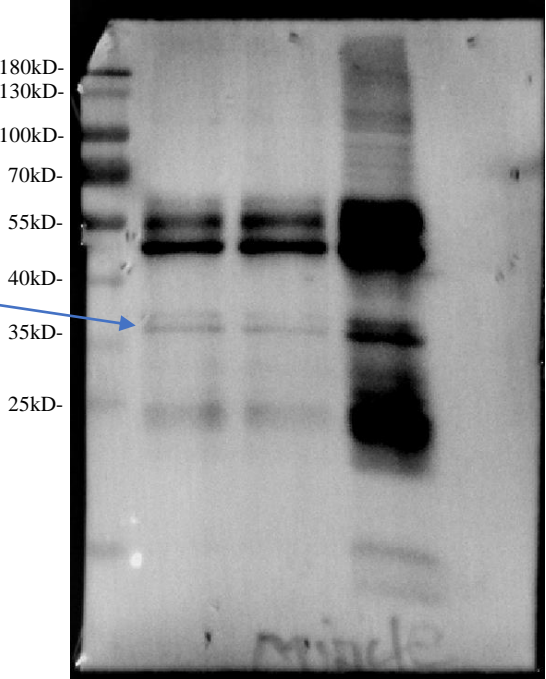

Fig6-H:

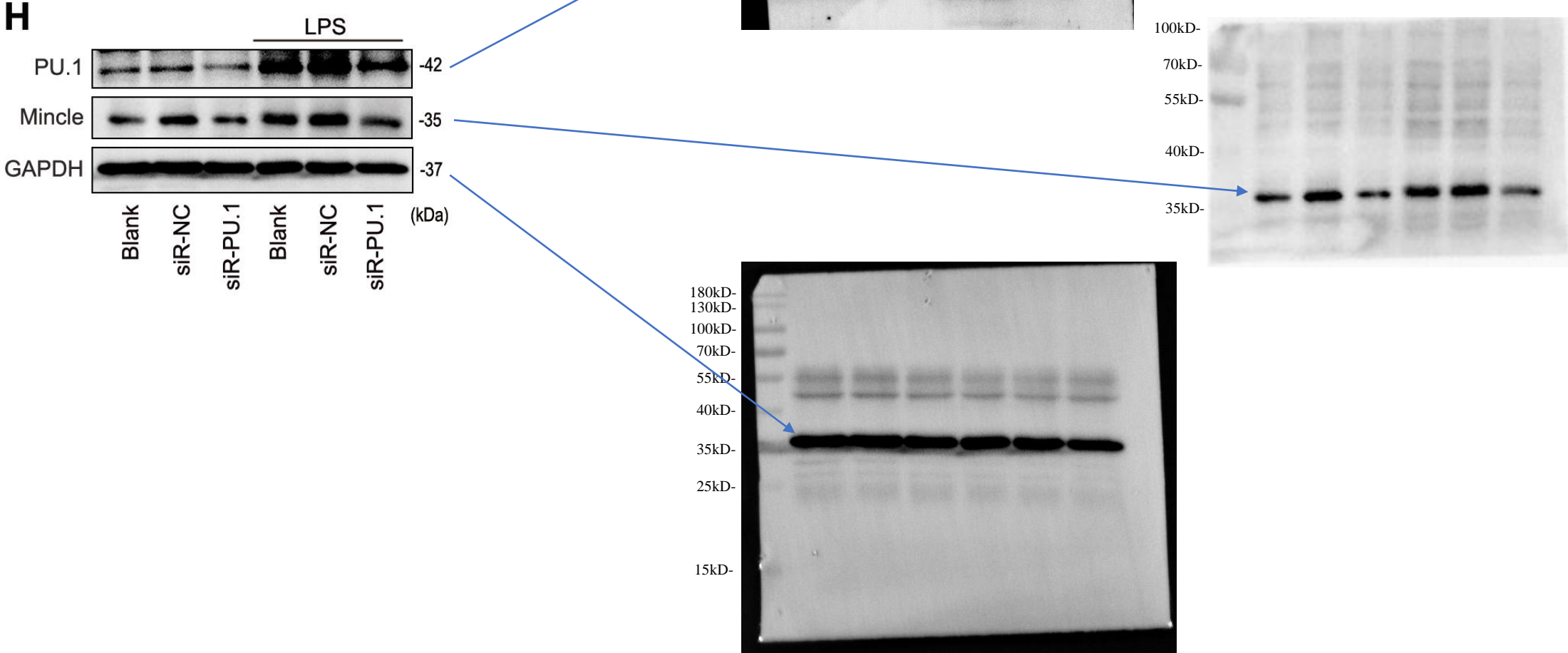

Fig7-D:

D

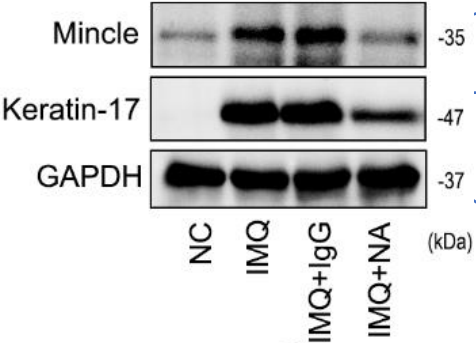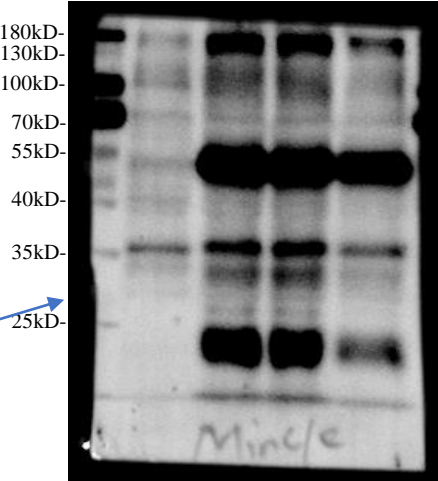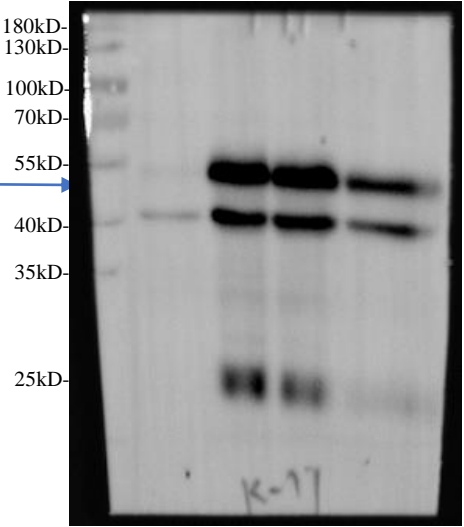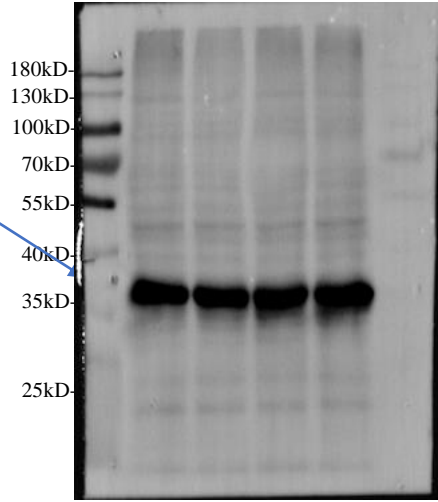

# E

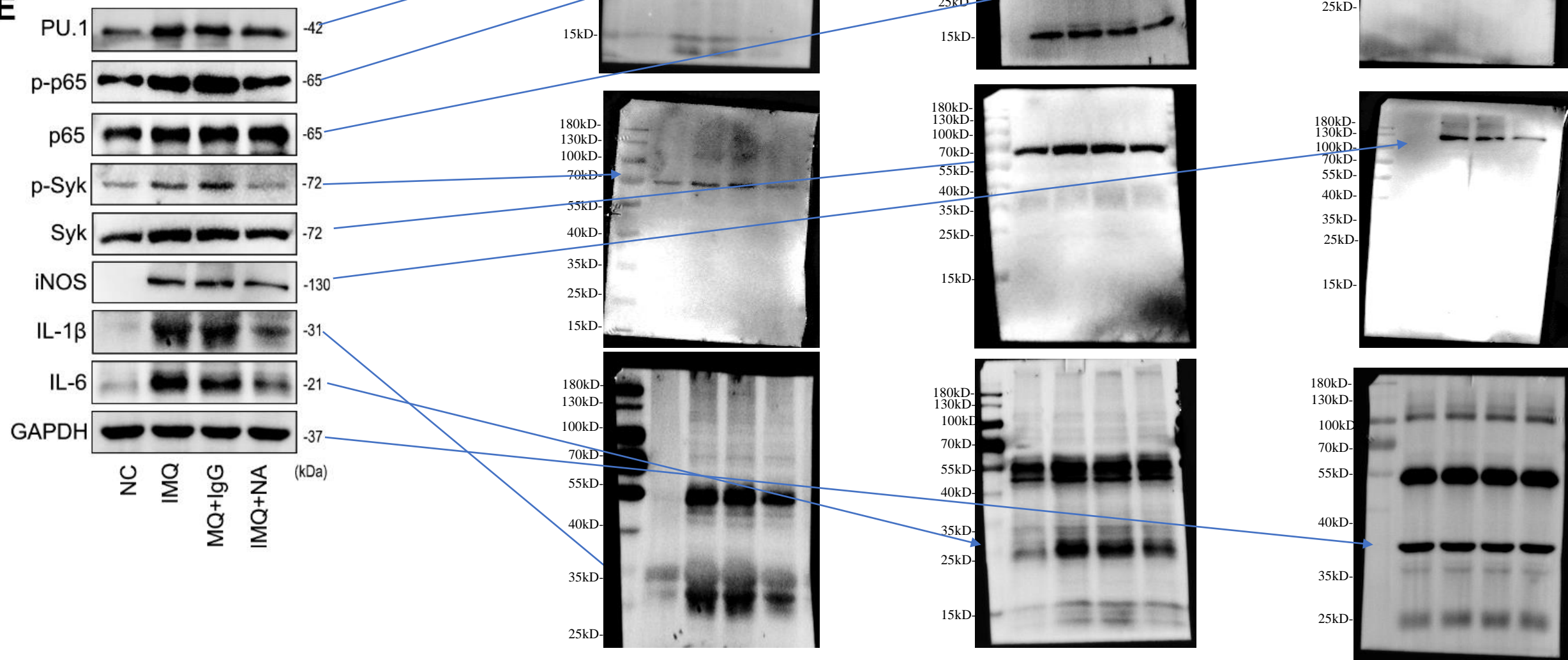

Supplement: Supplementary file 2 — WB RAW DATA [file 41420_2023_1444_MOESM2_ESM.pdf]
